# Increased chloroplast area in the rice bundle sheath through cell-specific perturbation of brassinosteroid signaling

**DOI:** 10.1093/plphys/kiaf108

**Published:** 2025-04-02

**Authors:** Lee Cackett, Leonie H Luginbuehl, Ross-William Hendron, Andrew R G Plackett, Susan Stanley, Lei Hua, Na Wang, Steven Kelly, Julian M Hibberd

**Affiliations:** Department of Plant Sciences, University of Cambridge, Cambridge CB2 3EA, UK; Department of Plant Sciences, University of Cambridge, Cambridge CB2 3EA, UK; Department of Plant Sciences, University of Oxford, South Parks Road, Oxford OX1 3RB, UK; School of Biosciences, University of Birmingham, Edgbaston B15 2TT, UK; Department of Plant Sciences, University of Cambridge, Cambridge CB2 3EA, UK; Department of Plant Sciences, University of Cambridge, Cambridge CB2 3EA, UK; Department of Plant Sciences, University of Cambridge, Cambridge CB2 3EA, UK; Department of Plant Sciences, University of Oxford, South Parks Road, Oxford OX1 3RB, UK; Department of Plant Sciences, University of Cambridge, Cambridge CB2 3EA, UK

## Abstract

In the leaves of C_3_ species such as rice (*Oryza sativa*), mesophyll cells contain the largest compartment of photosynthetically active chloroplasts. In contrast, plants that use the derived and more efficient C_4_ photosynthetic pathway have a considerable chloroplast compartment in both bundle sheath and mesophyll cells. Accordingly, the evolution of C_4_ photosynthesis from the ancestral C_3_ state required an increased chloroplast compartment in the bundle sheath. Here, we investigated the potential to increase chloroplast compartment size in rice bundle sheath cells by manipulating brassinosteroid signaling. Treatment with brassinazole, a brassinosteroid biosynthesis inhibitor, raised leaf chlorophyll content and increased the number but decreased the area of chloroplasts in bundle sheath cells. Ubiquitous overexpression of the transcription factor–encoding *BRASSINAZOLE RESISTANT 1* (*OsBZR1*) increased bundle sheath chloroplast area by up to 45%, but these plants became chlorotic. However, when *OsBZR1* expression was driven by a bundle sheath-specific promoter, the negative effects on growth and viability were alleviated while chloroplast area still increased. In summary, we report a role for brassinosteroids in controlling chloroplast area and number in rice and conclude that cell-specific manipulation of brassinosteroid signaling can be used to manipulate the chloroplast compartment in rice bundle sheath cells.

## Teaser

Cell-specific manipulation of brassinosteroid signaling reveals a role in chloroplast development in rice that can be used to increase chloroplast area in bundle sheath cells.

## Introduction

Increasing crop yield is considered imperative to feed a growing population, and improving photosynthetic efficiency is recognized as one possible approach to achieve this (Smith et al. 2023). In land plants, photosynthesis takes place in chloroplasts, the development of which is initiated by the perception of light and is modulated by various hormones (Cackett et al. 2022). This interplay allows the chloroplast content of each cell type to be tuned to the needs of the cell. For example, in C_3_ species such as rice (*Oryza sativa*), carbon fixation occurs primarily in mesophyll (M) cells that are densely packed with chloroplasts (Sage and Sage 2009). Although bundle sheath (BS) cells in C_3_ plants also contain chloroplasts, the proportion of cell volume they occupy is much lower than in M cells (Sage and Sage 2009). In contrast, C_4_ plants such as maize (*Zea mays*) contain a greatly enhanced chloroplast volume in BS cells (Lee et al. 2023). This allows photosynthetic reactions to be partitioned between the M and BS such that a biochemical pump concentrates CO_2_ in BS cells where RuBisCO accumulates. This C_4_ cycle reduces oxygenation of RuBisCO and the subsequent photorespiratory reactions, enabling photosynthetic efficiency to be increased by up to 50% (Sage et al. 2012). Understanding the differences in chloroplast biogenesis between cell types is therefore relevant to attempts to engineer C_3_ leaves such that they operate a C_4_-like photosynthesis.

Chloroplast biogenesis is primarily controlled by transcriptional regulators belonging to the *GOLDEN2-LIKE* (*GLK*), *GATA NITRATE-INDUCIBLE CARBONMETABOLISM-INVOLVED* (*GNC*), and *CYTOKININ RESPONSIVE GATA FACTOR 1* (*CGA1*) gene families (reviewed by Cackett et al. 2022). Recently, an additional regulator from the *RR-TYPE MYOBLASTOMA RELATED* (*RR-MYB*) gene family has been identified (Frangedakis et al. 2024), but it is not yet known how it responds to signals inducing chloroplast biogenesis, nor whether other transcription factors are involved. Overexpression of *GLK*s in multiple species increases chlorophyll and chloroplast production and can stimulate this in tissues that normally contain a very limited chloroplast compartment (Kobayashi et al. 2012, 2013; Nakamura et al. 2009; Wang et al. 2017). Constitutive overexpression of *ZmG2* in rice grown in the field increased photosynthesis, vegetative biomass, and grain yield (Li et al. 2020). Overexpression of *GNC* and *CGA1* increased chloroplast planar area in Arabidopsis (*Arabidopsis thaliana*) (Hudson et al. 2011) and rice BS cells (Hudson et al. 2013; Lee et al. 2021; Lim et al. 2024). However, neither overexpression of *GLK* or *CGA1* stimulated chloroplast biogenesis in the rice BS to the extent that their chloroplast content matched that of C_4_ sorghum (*Sorghum bicolor*) or maize. While unknown regulators may control the enhanced biogenesis of BS chloroplasts in C_4_ species, it is also plausible that known components initiating this process are responsible, but the complete network of control has not yet been elucidated.

The brassinosteroid (BR) signaling pathway acts to repress chlorophyll accumulation and chloroplast biogenesis in the dark and is inhibited after light is perceived and de-etiolation is induced. In the dark, BRs act with PHYTOCRHOME INTERACTING FACTORS (PIFs) to negatively regulate photosynthesis gene expression (Oh et al. 2012). Upon exposure to light, PIFs are degraded in response to phytochrome signaling and the induction of *GNC* expression represses BR signaling, allowing activation of chloroplast biogenesis (reviewed by Cackett et al. 2022). In the dark, Arabidopsis BR-related mutants such as *deetiolated2 (det2)*, *dwarf4 (dwf4)*, *constitutive photomorphogenic dwarf (cpd)*, *brassinosteroid-insensitive1 (bri1)*, and *brassinosteroid-insensitive 2* (*bin2)* show characteristics of de-etiolation including differentiated chloroplasts, short hypocotyls, development of true leaves, and expression of light-regulated genes (Chory et al. 1991; Clouse et al. 1996; Szekeres and Né 1996; Azpiroz et al. 1998; Li et al. 2001; Tachibana et al. 2022). The transcription factor BRASSINAZOLE RESISTANT 1 (BZR1) mediates the BR-modulated negative control of photomorphogenesis by repressing genes involved in light signaling and chloroplast development including photoreceptors such as PHOTOTROPIN 1; transcription factors such as *GATA2*, *GATA4*, and *GLK1&2*; and photosynthesis genes associated with chlorophyll biosynthesis (Luo et al. 2010; Sun et al. 2010; Yu et al. 2011; Wang et al. 2020). It is thought that the BZR1-mediated repression of chlorophyll biosynthesis avoids overaccumulation of protochlorophyllide in the dark so that when light is perceived, photooxidative damage is minimized and greening promoted (Wang et al. 2020).

The role of BRs and BZR1 during de-etiolation, organ development, cell elongation, and chlorophyll accumulation is well documented in Arabidopsis and, to a lesser extent, in rice. However, to our knowledge, there are no analyses in rice demonstrating if or how BRs control chloroplast biogenesis in terms of size and number per cell. We therefore assessed how pharmacological and genetic perturbations to BR signaling affect the planar area and number of chloroplasts in the BS of rice. Our analysis indicated that BRs alter the number and area of chloroplasts in the rice BS during de-etiolation and at later stages of development. Ubiquitous overexpression of *OsBZR1* resulted in larger chloroplasts in the BS but had adverse effects on plant health and yield. However, when overexpression of *OsBZR1* was driven by a BS-specific promoter, increased BS cell chloroplast area was maintained while the adverse effects on growth mitigated. Overall, these data are consistent with an approach in which cell-specific manipulation of BR signaling could be used to manipulate chloroplast number and size in rice.

## Results

### BRs modulate chloroplast size and number in the rice bundle sheath

**Figure 1.**
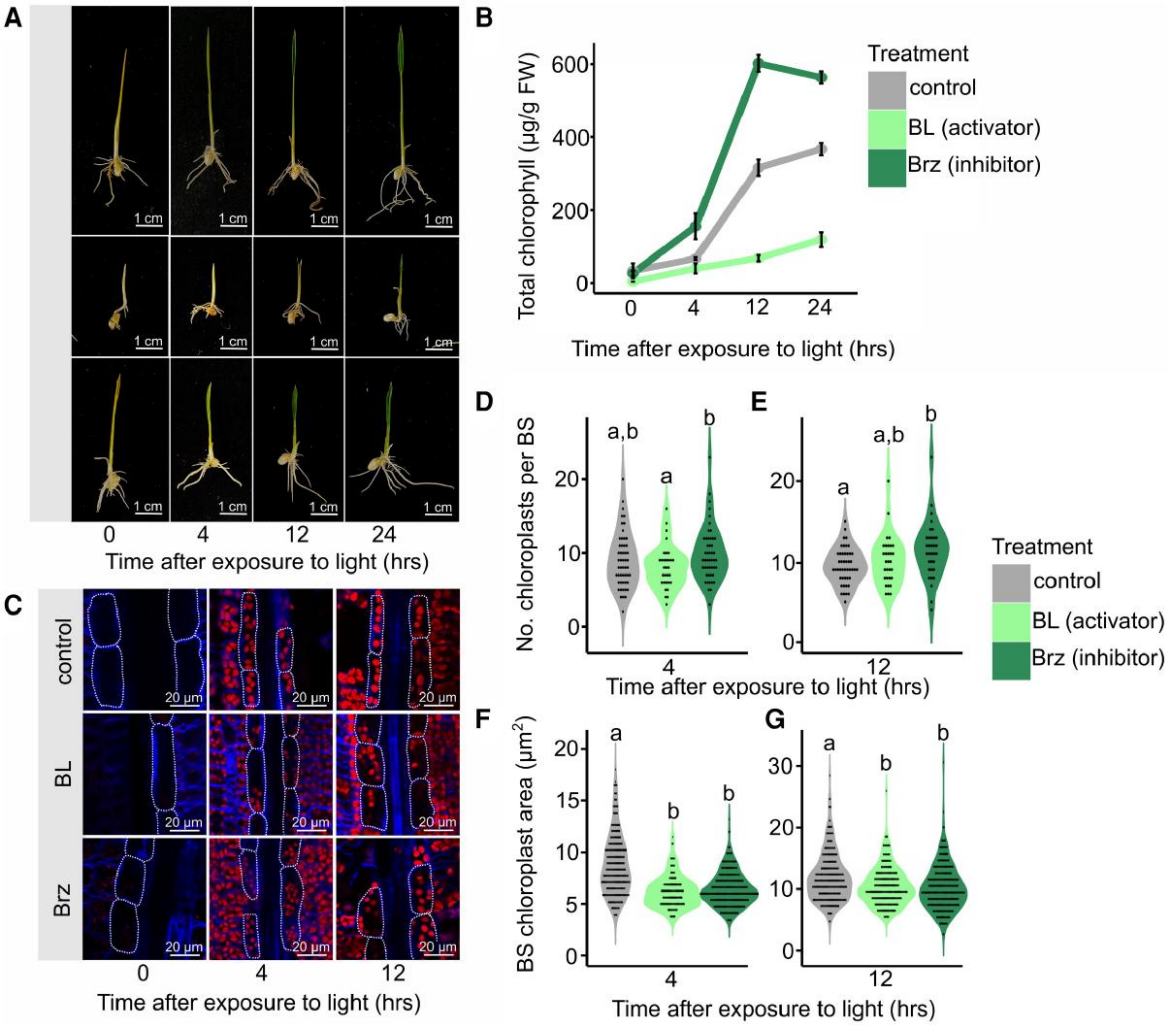
BRs modulate chlorophyll accumulation and BS chloroplast area and number during de-etiolation. Seeds were germinated in water and transferred in the dark to ½ MS agar media with or without 10 *µ*M Brassinolide (BL) or 10 *µ*M Brassinazole (Brz). After 4 d, seedlings were transferred to light and shoot tissue harvested 0, 4, 12, and 24 h later for chlorophyll quantification and imaging using confocal laser scanning microscopy. **A)** Representative images of control and BL/Brz-treated seedlings during de-etiolation. **B)** Mean chlorophyll content during de-etiolation normalized to amount of fresh weight harvested. Data are from 5 biological repeats for each time point in each treatment. Error bars represent standard error. **C)** Confocal images of BS cells and chloroplasts during de-etiolation. Red and blue channels indicate chloroplasts and cell walls, respectively. Dotted lines highlight BS cells. **D** and **E)** Number of chloroplasts per BS cell in control and BL/Brz-treated seedlings 4 h **D)** and 12 h **E)** after exposure to light. Data are derived from confocal microscopy and from at least 30 BS cells for each time point in each treatment. **F** and **G)** BS cell chloroplast area in control and BL/Brz-treated seedlings 4 h **F)** and 12 h **G)** after exposure to light. Data are derived from confocal microscopy and from at least 150 chloroplasts for each time point in each treatment. For **D)** to **G)**, individual data points are overlaid as dots, where each dot represents a single observation. Dots are stacked symmetrically along the *y* axis to visualize data density within each group. Letters above violins represent statistically significant differences (*P* ≤ 0.05) in mean values as determined by Fisher Lsd post hoc analysis following a 1-way ANOVA.

To initiate an understanding of the role of BRs in modulating chloroplast biogenesis in rice, we applied the active BR, brassinolide (BL), or the biosynthesis inhibitor, brassinazole (Brz), to seedlings during de-etiolation. For this, seeds were germinated for 2 d in the dark, transferred to BL- or Brz-containing media for 4 d in the dark, and then exposed to light for 0, 4, and 12 h. In control plants, greening and expansion of the first leaf were evident after exposure to light as expected (Fig. 1A). Consistent with previous analyses (Hong et al. 2002; Mori et al. 2002), BL treatment inhibited de-etiolation such that rice seedlings showed reduced leaf expansion and greening (Fig. 1A; Supplementary Fig. S1). Quantification of whole seedling chlorophyll levels confirmed that its accumulation was reduced compared with controls (Fig. 1B). In contrast to the BL treatment, seedlings treated with Brz showed an increase in accumulation of chlorophyll after exposure to light compared with controls (Fig. 1B). Chloroplast content in the BS cells of the first leaf of controls and treated seedlings was imaged using confocal laser scanning microscopy at 0, 4, and 12 h after light exposure (Fig. 1C). Brz treatment resulted in more chloroplasts per BS cell compared with controls after 12 h of light, whereas BL treatment produced no significant difference (Fig. 1, D and E). Both BL and Brz treatments resulted in smaller BS chloroplasts compared to control plants, in terms of mean planar area (Fig. 1, F and G). BS cell size decreased compared to the control as a result of the BL treatment and was unchanged in the Brz treatment compared with the control (Supplementary Fig. S2). Overall, these data are consistent with previous studies from other species reporting that BRs modulate chlorophyll accumulation during de-etiolation but also indicate that BRs can control chloroplast number and planar area.

**Figure 2.**
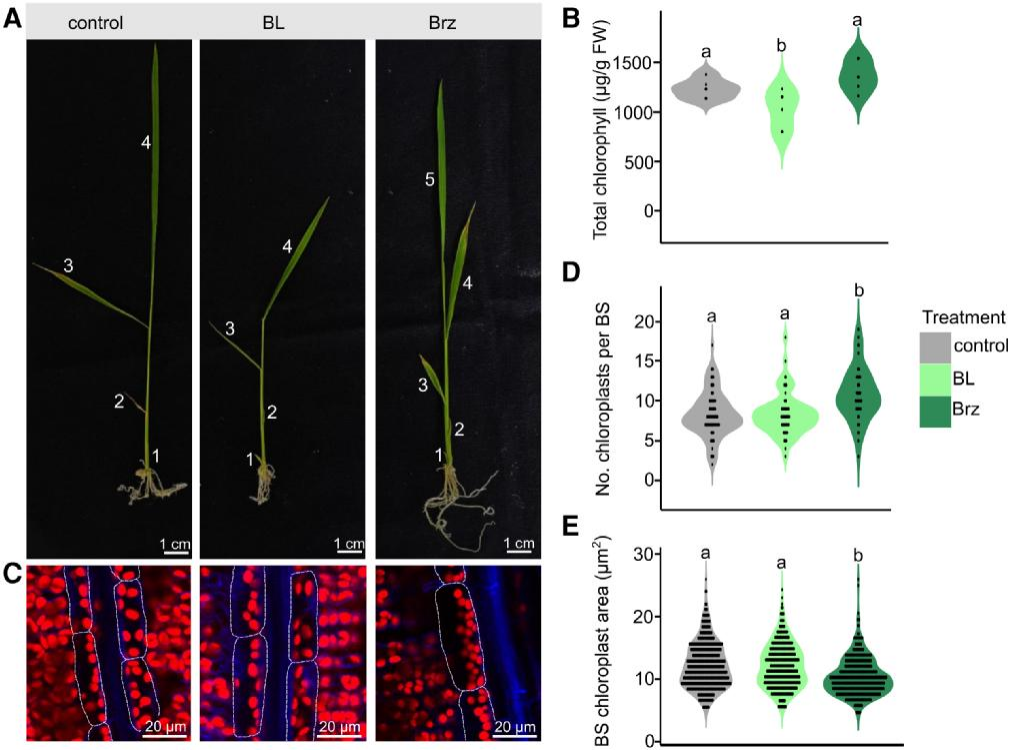
BRs modulate chlorophyll accumulation and BS chloroplast area and number in mature leaves. Seeds were germinated in water and transferred to ½ MS agar media with or without 10 *µ*M Brassinolide (BL) or 10 *µ*M Brassinazole (Brz). Leaf 4, once fully expanded, was harvested for chlorophyll quantification and imaging using confocal laser scanning microscopy. **A)** Representative images of control and BL/Brz-treated plants. Numbers indicate leaf number in order of appearance. **B)** Chlorophyll content of Leaf 4 from control and BL/Brz-treated plants. Data are from 6 biological repeats for each treatment normalized to amount of fresh weight harvested. **C)** Confocal images of BS cells and chloroplasts in Leaf 4 of controls and BL/Brz-treated plants. Red and blue channels indicate chloroplasts and cell walls, respectively. Dotted lines highlight BS cells. **D)** Number of chloroplasts per BS cell in Leaf 4 from controls and BL/Brz-treated plants. Data are derived from confocal microscopy and from at least 70 BS cells for each treatment. **E)** BS cell chloroplast area in Leaf 4 from controls and BL/Brz-treated plants. Data are derived from confocal microscopy and from at least 550 chloroplasts for each time point in each treatment. For **B)**, **D)**, and **E)**, individual data points are overlaid as dots, where each dot represents a single observation. Dots are stacked symmetrically along the *y* axis to visualize data density within each group. Letters above violins represent statistically significant differences (*P* ≤ 0.05) in mean values as determined by Fisher Lsd post hoc analysis following 1-way ANOVA.

To determine whether changes to chloroplast number and size were maintained later in development, plants were grown in BL or Brz and Leaf 4 harvested once it was fully expanded. Both treatments impacted overall plant growth and development. For example, plants treated with BL developed the same number of leaves as controls, but leaf length was reduced (Fig. 2A). Brz caused faster development such that more leaves were evident (Fig. 2A), and they contained more veins than controls (Supplementary Fig. S3). Consistent with the initial de-etiolation experiments, chlorophyll content of Leaf 4 was reduced by the addition of BL (Fig. 2B) while it had no significant impact on chloroplast area and number in the BS at this developmental stage (Fig. 2, C to E). Conversely, Brz treatment increased BS chloroplast number and decreased chloroplast area compared with controls (Fig. 2, C to E) but with no change in chlorophyll content (Fig. 2B). Overall, these results indicate that Brz, an inhibitor of BR biosynthesis, modulates the area and number of chloroplasts in rice BS cells both during de-etiolation and at later stages of leaf development.

### Ubiquitous overexpression of OsBZR1 increases chloroplast area in BS cells but has adverse effects on plant growth

Given that perturbation of BR signaling through exogenous treatments gave rise to changes in chloroplast area and number in rice BS cells, we sought to determine whether analogous changes could be achieved through genetic activation of BR-responsive gene expression. As BZR1 is the primary transcription factor that mediates BR-responsive gene expression in Arabidopsis (Luo et al. 2010; Sun et al. 2010; Yu et al. 2011; Wang et al. 2020), we chose to investigate whether manipulation of the expression of the orthologous regulatory gene in rice could achieve the desired changes in chloroplast development. Phylogenetic interrogation of the BZR1 gene family revealed that there is a single gene in rice (LOC_Os07g39220) that is putatively orthologous (i.e. equally related) to BZR1, BES1 (BZR2), BEH1, and BEH2 in Arabidopsis (Supplementary Fig. S4), with no other rice gene homologs in this same clade. Accordingly, we hypothesized that this single gene is likely the primary transcription factor that mediates BR-responsive gene expression in rice. Moreover, previous investigations had implicated this gene in BR signaling in rice leading to the naming of the gene *OsBZR1* (Bai et al. 2007). Thus, both overexpression and RNA interference (RNAi) lines were generated to alter the expression of Os*BZR1* in the rice leaf.

**Figure 3.**
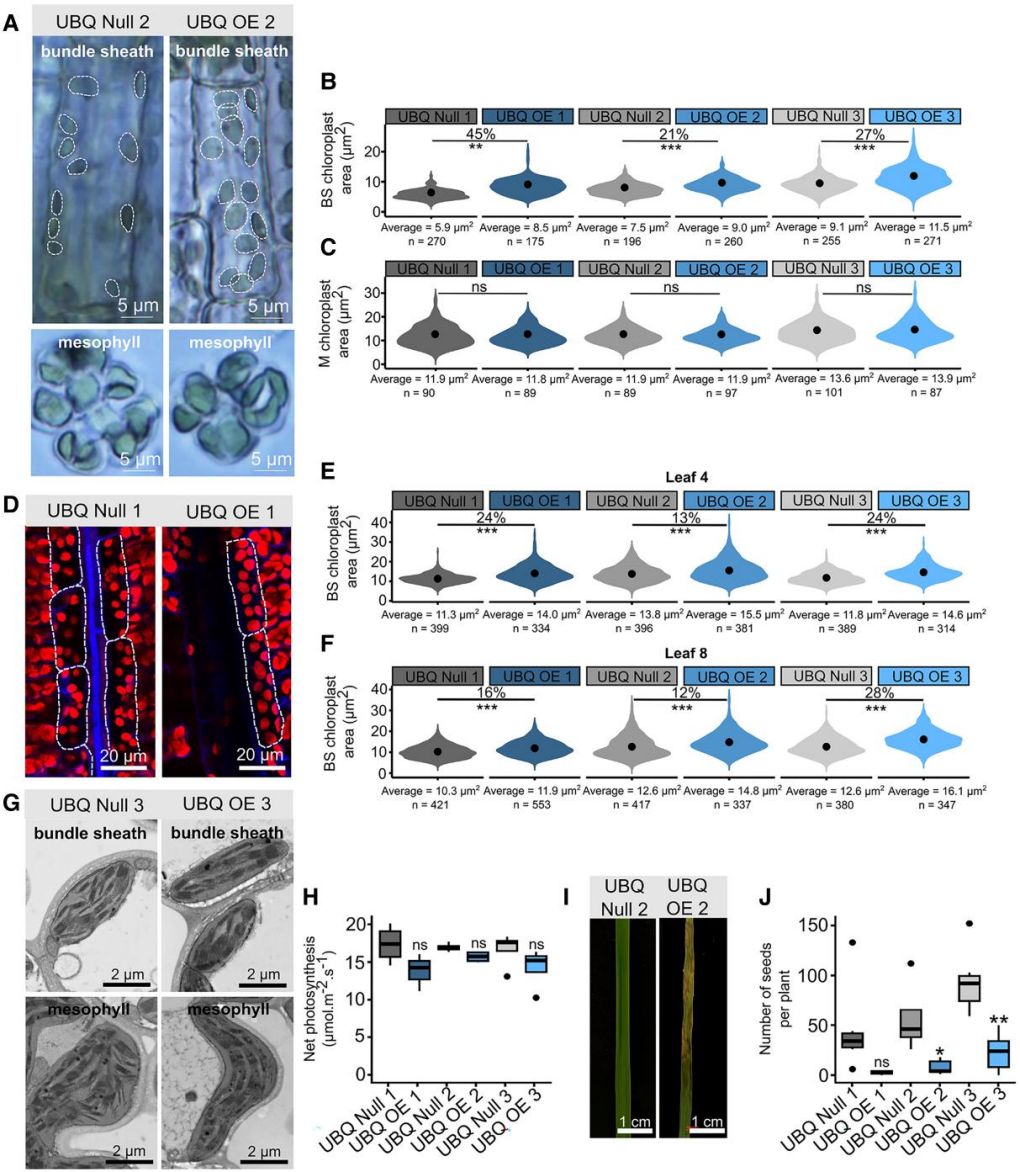
Ubiquitous overexpression of *OsBZR1* increases the area of chloroplasts in rice bundle sheath cells but impacts plant health. The rice codon-optimized sequence for *BZR1* (rco*OsBZR1*) was cloned upstream of the maize *UBIQUITIN* promoter (p*ZmUBI*) and transformed into rice to generate constitutive overexpression lines (UBQ OE). **A)** Representative images from brightfield microscopy of individual BS and M cells from Leaf 8. Dotted lines highlight individual chloroplasts within each BS cell. **B** and **C)** Chloroplast area in BS **B)** and M **C)** cells from Leaf 8. Chloroplast areas are from quantification of the brightfield microscopy. The center dot within each violin represents the mean chloroplast area. Percentages above violins indicate the change in chloroplast area of overexpressor lines compared with corresponding nulls. No statistically significant change in chloroplast area is represented by “ns”. The value below each violin is the mean chloroplast area calculated for that line, and *n* represents the number of chloroplasts quantified. Four biological replicates were used for each line. **D)** Images derived from confocal laser scanning microscopy of BS cells and chloroplasts in Leaf 4. Red and blue channels indicate chloroplasts and cell walls, respectively. Dotted lines highlight BS cells. **E** and **F)** BS cell chloroplast area in Leaf 4 **E)** and Leaf 8 **F)**. Chloroplasts quantified are from confocal microscopy. The center dot within each violin represents the mean chloroplast area. Percentages above violins indicate the change in chloroplast area of overexpressor lines compared with corresponding nulls. No statistically significant change in chloroplast area is represented by “ns”. The value below each violin is the mean chloroplast area calculated for that line, and *n* represents the number of chloroplasts assessed. Four biological replicates were used for each line. **G)** SEM images of BS and M cell chloroplasts from Leaf 8. **H)** Rate of net photosynthesis under conditions of growth for overexpressor lines compared with corresponding nulls. Data are from 4 biological replicates for each line. **I)** Representative images of Leaf 8 from null and overexpressor plants of the same age depicting increased senescence. **J)** Number of seeds produced by each line. Data are from 4 biological replicates for each line. For **B)**, **C)**, **E)**, **F)**, **H)**, and **J)**, stars above violins or boxes indicate a statistically significant difference between overexpressor lines compared with corresponding null as determined by independent *t*-test, where *P* ≤ 0.05 is flagged with 1 star (*), *P* ≤ 0.01 is flagged with 2 stars (**), and *P* ≤ 0.001 is flagged with 3 stars (***). No statistically significant change is represented by “ns”. For **H)** and **J)**, the box plots show the median and the interquartile range between the first and third quartiles and whiskers indicate the smallest and largest values within 1.5 × interquartile range from the quartiles, while points beyond this range are considered outliers.

No reduction in transcript abundance was detected in T_2_  *OsBZR1* RNAi plants (Supplementary Fig. S5B), and so phenotyping was not undertaken. However, ubiquitous overexpression using the maize UBIQUITIN promoter was successful. Here, 3 independent homozygous single-copy transgenic lines along with their respective null segregants (lines which had been through the transformation process but do not contain the genetic modification themselves) were identified (Supplementary Fig. S6, A and B). Reverse transcription quantitative PCR (RT-qPCR) and bulk RNA sequencing on T_2_ plants was conducted to confirm the level of endogenous *OsBZR1* and transgene expression (Supplementary Fig. S6, C to E). Hereafter, these lines are referred to as UBQ Null 1, UBQ OE 1, UBQ Null 2, UBQ OE 2, UBQ Null 3, and UBQ OE 3. Brightfield microscopy was used to image isolated BS and M cells from fully expanded Leaf 8 (Fig. 3A). The individual chloroplast planar area was significantly larger for all 3 overexpression lines compared with the respective nulls in BS cells (Fig. 3B). UBQ OE 1 showed the largest effect with individual chloroplast area being increased by 45% (Fig. 3B). The extent to which chloroplast area increased corresponded with the degree of *OsBZR1* overexpression within each line (Supplementary Fig. S6C). There were no statistically significant differences in chloroplast area in M cells in any UBQ OE line (Fig. 3C). To confirm these findings with a higher throughput approach (Billakurthi and Hibberd 2023) allowing a larger number of chloroplasts to be assessed, we next used confocal laser scanning microscopy (Fig. 3D). Consistent with the analysis of single cells after brightfield microscopy, this showed that overexpression of *OsBZR1* increased individual chloroplast area in BS cells in Leaf 4 and Leaf 8 compared with corresponding null lines (Fig. 3, E and F). The number of chloroplasts per BS cell in Leaf 4 or Leaf 8 was calculated. Although there was a statistically significant difference in chloroplast number between null and UBQ overexpressor lines when Leaf 4 was assessed, the absolute values were 10 and 8 chloroplasts per cell respectively, and so this difference was small. Furthermore, there were no statistically significant differences when Leaf 8 was assessed (Supplementary Fig. S7, A and B). Scanning electron microscopy (SEM) detected no evident changes in BS chloroplast ultrastructure between null and overexpression lines (Fig. 3G). There were no consistent differences in whole leaf chlorophyll content (Supplementary Fig. S8), and only 1 of the 3 UBQ OE lines showed a significant difference in BS cell size compared with the corresponding null line (Supplementary Fig. S9, A and B). The rate of net photosynthesis in young fully expanded leaves was not affected by ubiquitous overexpression of *OsBZR1* (Fig. 3H). Notably, the UBQ OE leaves senesced rapidly soon after maturation (Fig. 3I), and the number of seeds produced by all UBQ OE plants was significantly lower than corresponding nulls (Fig. 3J). We therefore sought to test whether a more targeted misexpression of *OsBZR1* in the rice BS could maintain chloroplast development without inducing premature senescence and decreased yield.

### Overexpression of OsBZR1 in the BS increases chloroplast area without inducing premature senescence

**Figure 4.**
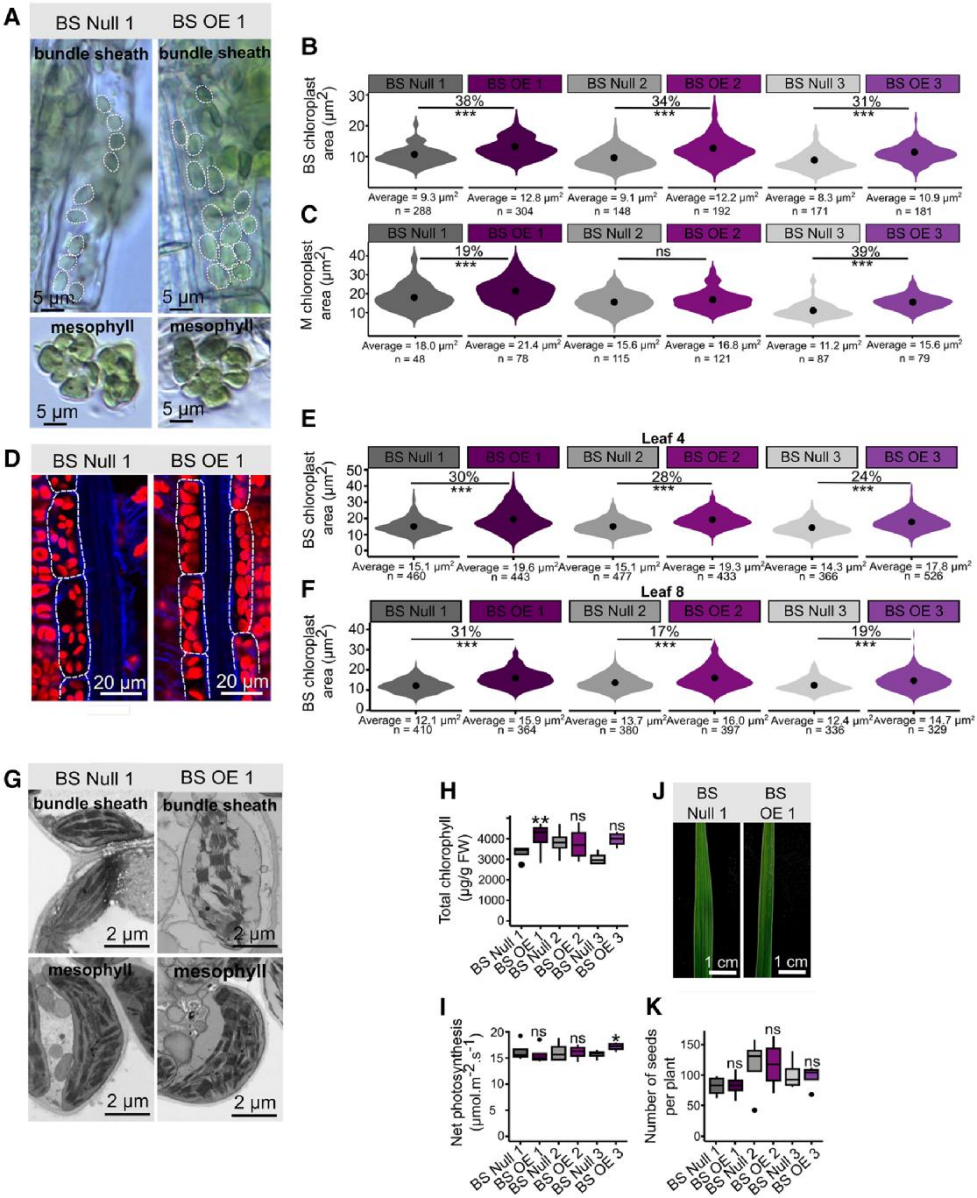
BS cell-specific overexpression of *OsBZR1* increases the area of chloroplasts in rice bundle sheath cells with no impact on plant health. The rice codon-optimized sequence for *BZR1* (rco*OsBZR1*) was cloned upstream of the rice BS cell-specific *SULPHITE REDUCTASE* promoter (p*OsSiR*) and transformed into rice to generate cell-specific overexpression lines (BS OE). **A)** Representative images from brightfield microscopy of individual BS and M cells from Leaf 8. Dotted lines highlight individual chloroplasts within each BS cell. **B** and **C)** Chloroplast area in BS **B)** and M **C)** cells from Leaf 8. Chloroplast areas are from quantification of the brightfield microscopy. The center dot within each violin represents the mean chloroplast area. Percentages above violins indicate the change in chloroplast area of overexpressor lines compared with corresponding nulls. No statistically significant change in chloroplast area is represented by “ns”. The value below each violin is the mean chloroplast area calculated for that line, and *n* represents the number of chloroplasts quantified. Four biological replicates were quantified for each line. **D)** Images derived from confocal laser scanning microscopy of BS cells and chloroplasts in Leaf 4. Red and blue channels indicate chloroplasts and cell walls, respectively. Dotted lines highlight BS cells. **E** and **F)** BS cell chloroplast area in Leaf 4 **E)** and Leaf 8 **F)**. Chloroplasts quantified are from the confocal microscopy. The center dot within each violin represents the mean chloroplast area. Percentages above violins indicate the change in chloroplast area of overexpressor lines compared with corresponding nulls. No statistically significant change in chloroplast area is represented by “ns”. The value below each violin is the mean chloroplast area calculated for that line, and *n* represents the number of chloroplasts quantified. Four biological replicates were used for each line. **G)** SEM images of BS and M cell chloroplasts from Leaf 8. **H)** Total chlorophyll in Leaf 8 of overexpressor lines compared with corresponding nulls. Data are from 4 biological repeats for each line. **I)** Rate of net photosynthesis under conditions of growth for overexpressor lines compared with corresponding nulls. Data are from 4 biological replicates for each line. **J)** Representative images of Leaf 8 from null and overexpressor plants of the same age. **K)** Number of seeds produced by each line. Data are from 4 biological replicates for each line. For **B)**, **C)**, **E)**, **F)**, **H)**, **I)**, and **K)**, stars above violins or boxes indicate a statistically significant difference between overexpressor and corresponding null as determined by independent *t*-test, where *P* ≤ 0.05 is flagged with 1 star (*), *P* ≤ 0.01 is flagged with 2 stars (**), and *P* ≤ 0.001 is flagged with 3 stars (***). No statistically significant change is represented by “ns”. For **H)**, **I)**, and **K)**, the box plots show the median and the interquartile range between the first and third quartiles and whiskers indicate the smallest and largest values within 1.5 × interquartile range from the quartiles, while points beyond this range are considered outliers.

The promoter of the rice *SULPHITE REDUCTASE* (*SIR*) gene generates strong expression in BS cells (Hua et al. 2024), and so we used it to drive expression of rco*OsBZR1* (Supplementary Fig. S10A). Three independent homozygous and single-copy overexpression lines (Supplementary Fig. S10B) along with corresponding null segregants were identified. RT-qPCR and bulk RNA sequencing on T_2_ plants was performed to confirm the level of endogenous *OsBZR1* and transgene expression (Supplementary Fig. S10, C to E). Hereafter, these are referred to as BS Null 1, BS OE 1, BS Null 2, BS OE 2, BS Null 3, and BS OE 3. As with the ubiquitous overexpressor, individual M and BS cells from the BS Null and OE lines were isolated and brightfield microscopy used to quantify chloroplast planar area (Fig. 4, A to C). All 3 overexpression lines contained larger BS cell chloroplasts when compared with corresponding null lines (Fig. 4B). Interestingly, chloroplast areas in M cells were also increased compared with the corresponding nulls in 2 out of the 3 lines (Fig. 4C). This may be due to very low levels of basal expression from the *SiR* promoter in M cells, or a noncell autonomous response whereby changes in 1 cell-type impact other cells. Analysis of BS cells from Leaf 4 and Leaf 8 by confocal laser scanning microscopy also indicated that BS cell chloroplasts were larger in BS OE plants compared with nulls (Fig. 4, D to F). There were no significant changes in the number of chloroplasts per BS cell in Leaf 4 or Leaf 8 in the BS OE lines compared with corresponding nulls (Supplementary Fig. S11, A and B). No clear differences in chloroplast ultrastructure between BS null and BS OE lines were discernible from SEM (Fig. 4G), nor could we detect differences in BS cell size (Supplementary Fig. S12, A and B). To initiate an understanding of whether increased chloroplast area was due to *OsBZR1* driving BR-related changes rather than perturbing other signaling pathways, the BS null and BS OE lines were grown in Brz which inhibits BR biosynthesis. For 2 of the 3 BS OE lines, the positive effect of BS cell-specific overexpression of *OsBZR1* was suppressed by Brz application (Supplementary Fig. S13). These data imply, but do not unequivocally prove, that BZR1 is acting via the BR signaling pathway to modify chloroplast area, and so further work will be needed to fully understand the mode of action of BZR1 in rice in the future.

Despite the statistically significant increase in BS chloroplast area, neither chlorophyll content nor rate of photosynthesis was consistently increased by cell-specific overexpression of *OsBZR1* (Fig. 4, H and I). However, unlike the ubiquitous overexpressors, the leaves of the BS OE plants did not show premature senescence (Fig. 4J), and the number of seeds produced per plant did not differ from controls (Fig. 4K). Overall, these data confirm that increasing *OsBZR1* expression can increase chloroplast area in rice BS cells, but also that cell-specific perturbation avoided deleterious effects on growth.

### Ubiquitous overexpression of OsBZR1 perturbs stress-, BR-, and hormone-related pathways while BS cell-specific overexpression does not

**Figure 5.**
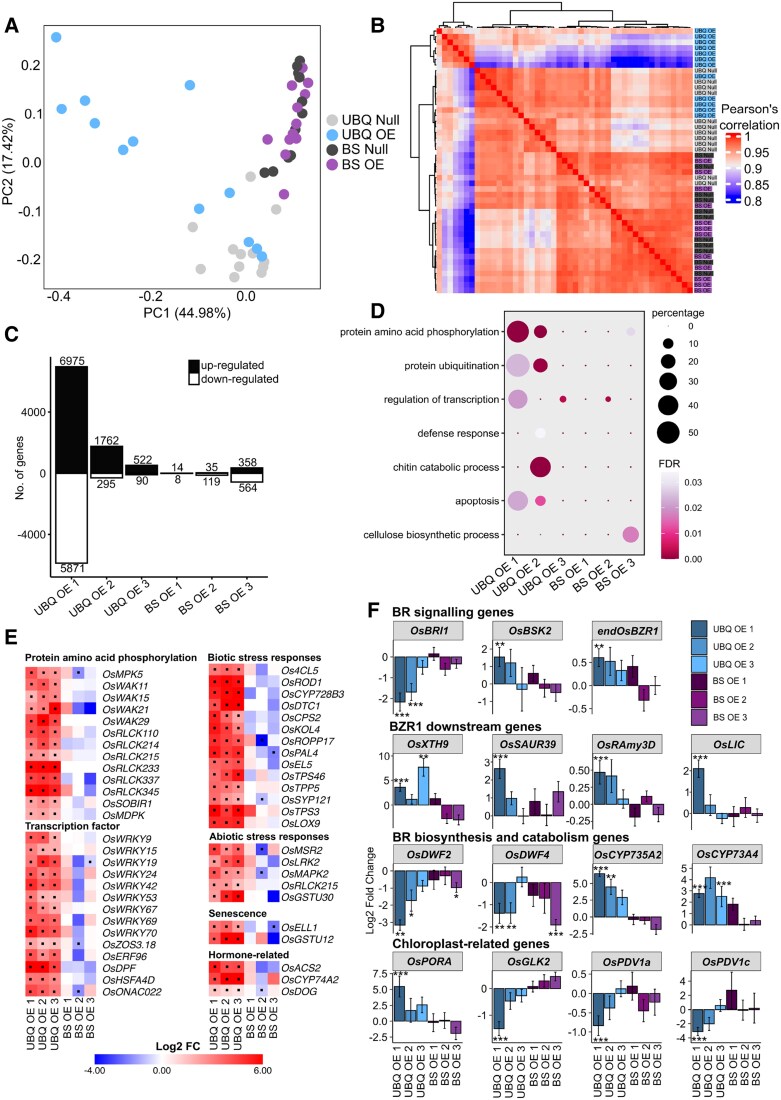
Transcriptome analyses of constitutive and BS cell-specific *OsBZR1* overexpressors. RNA from Leaf 4 of 4 biological replicates from all of the UBQ Null, UBQ OE, BS Null, and BS OE lines was used for cDNA library construction and subsequent transcriptome sequencing. **A)** PCA indicates the transcriptome data separated primarily based on genotype. **B)** Heatmap showing relatedness of all samples based on Pearson's correlation performed on log transformed data. The color of each block indicates the Pearson's correlation value. **C)** Numbers of significantly (*P*-adj. < 0.05) DEGs were determined by DESeq2 analysis which compared samples from an overexpressor with its corresponding null. Upregulated genes are represented by black bars and downregulated genes by white bars. Numbers above each bar indicate the count of DEGs represented by the bar. **D)** GO terms enriched in the significantly DEG lists from the overexpressors. Circle size depicts percentage, which is the number of genes in the given DEG list with the GO term divided by the number of genes in the reference genome with the GO term. The color of each circle indicates the statistical significance of enrichment for each GO term, with FDR representing false discovery rate. **E)** Heatmaps showing the expression of genes of interest significantly differentially expressed in all 3 lines of constitutive overexpressors. The color of each block indicates the Log2 fold change. A dot within each block indicates a statistically significant change in Log2 fold change. **F)** Log2 fold change in expression of genes involved in BR signaling, BZR1 downstream genes, BR biosynthesis, and catabolism and chloroplast development. Stars above bars indicate a statistically significant difference in Log2 fold change expression where *P-*adj. ≤ 0.05 is flagged with 1 star (*), *P-*adj. ≤ 0.01 is flagged with 2 stars (**), and *P-*adj. ≤ 0.001 is flagged with 3 stars (***). Error bars represent standard error. For **E)** and **F)**, Log2 fold change was determined by DESeq2 analyses using 4 biological replicates for each line. Statistical significance was determined using the Wald test for pairwise comparisons and *P*-values adjusted for multiple testing using the Benjamini–Hochberg (false discovery rate) correction. Refer to Supplementary dataset S4 for full gene names and gene IDs.

To gain insight into how ubiquitous and cell-specific overexpression of *OsBZR1* reprogrammed overall gene expression, we conducted bulk RNA sequencing on mature Leaf 4 from UBQ Null, UBQ OE, BS Null, and BS OE plants. Principal component analysis (PCA) showed that ubiquitous overexpression of *OsBZR1* caused the most variance and impacted on both the first and second components (Fig. 5A). In contrast, when *OsBZR1* was expressed in the BS, there were few overall transcriptional changes, with samples from BS Null and BS OE clustering together (Fig. 5A). The trends in changes to transcript abundance were also evident from a heatmap derived from Pearson's correlation analysis (Fig. 5B). Notably, correlation in mRNA abundance was weakest between UBQ OE and corresponding null lines whereas BS OE lines had high correlation with the corresponding BS null lines (Fig. 5B). Transcripts whose abundance was significantly different were identified by comparing each overexpression line with the corresponding null. Only genes with statistically significant changes (i.e. adjusted *P* < 0.05) were retained for subsequent analyses. This identified 6,975, 1,762, and 522 significantly upregulated genes and 5,871, 295, and 90 genes downregulated genes in the 3 ubiquitous overexpressors (Fig. 5C; Supplementary Data Sets 1 and 2). Notably, the extent to which transcript abundance was perturbed corresponded to the degree of *OsBZR1* overexpression in these lines (Fig. 5C; Supplementary Fig. S6C). As would be expected from the PCA and Pearson's correlation analysis, overexpression of *OsBZR1* from the BS promoter generated limited alterations to transcript abundance with only 14, 35, and 358 significantly upregulated genes and 8, 119, and 564 significantly downregulated genes compared with null lines (Fig. 5C; Supplementary Data Sets 1 and 2). As with the ubiquitous overexpressor, the extent to which transcript abundance was perturbed corresponded to the degree of *OsBZR1* overexpression in these lines (Fig. 5C; Supplementary Fig. S10C).

Gene Ontology (GO) enrichment analyses on transcripts that were up- and downregulated identified several terms of interest, with the majority of these terms enriched in the ubiquitous overexpressing lines and not in the BS overexpressing lines (Fig. 5D; Supplementary Data Set 3). For example, GO terms for “protein amino acid phosphorylation”, “protein ubiquitination”, and “regulation of transcription” indicate a global change in transcription and protein degradation after ubiquitous *OsBZR1* overexpression. We note that several genes contributing to the GO terms involved in protein phosphorylation or transcription including 4 *Wall Associated Kinase* (*WAK*), 6 *Receptor-like Cytoplasmic Kinase* (*RLCK*), and 9 *WRKY* transcription factors were significantly upregulated in all 3 ubiquitous overexpressors, but this was not the case when *OsBZR1* expression was driven in the BS (Fig. 5E; Supplementary Data Set 4). Moreover, only the ubiquitous overexpressing line had enriched GO terms “defense response”, “chitin catabolic process”, and “apoptosis” that are indicative of biotic and/or abiotic stress responses and are consistent with the increased senescence observed in the ubiquitous overexpressors. Specific genes associated with biotic and abiotic stress responses and senescence were also significantly upregulated in ubiquitous overexpressors (Fig. 5E). Consistent with the lack of GO term enrichment in BS-specific *OsBZR1* lines, transcript abundance of the genes involved in biotic and abiotic stress responses and senescence was not altered (Fig. 5E; Supplementary Data Set 4). It was notable that transcripts derived from the *OsACS2*, *OsCYP74A2*, and *OsDOG* genes involved in ethylene, jasmonic acid, and gibberellic acid synthesis were more abundant in all 3 ubiquitous *OsBZR1* overexpression lines compared to corresponding nulls, indicating possible hormonal crosstalk had been induced (Fig. 5E; Supplementary Data Set 4). Again, these genes were not differentially expressed in the lines when *OsBZR1* was driven from the BS promoter. Interestingly, the only enriched GO term specific to the BS overexpressor was “cellulose biosynthetic process” (Fig. 5D).

We next examined the expression of genes involved in BR signaling, genes acting downstream of BZR1, or genes involved in BR biosynthesis and catabolism to better understand how overexpression of *OsBZR1* perturbed these processes (Fig. 5F; Supplementary Data Set 4). In ubiquitous overexpressors, the BR receptor *OsBRI1* was significantly downregulated while the BR signaling kinase *OsBSK2* and the endogenous *OsBZR1* gene were significantly upregulated (Fig. 5F; Supplementary Data Set 4). This was not evident when *OsBZR1* was overexpressed in the BS. Putative rice orthologs (*OsXTH9* and *OsSAUR39*) of Arabidopsis genes *AtTCH4* and *AtSAUR-AC1* that have been used as BR-induced marker genes (Xu et al. 1995; Iliev et al. 2002; Nakamura et al. 2003) were significantly upregulated in the constitutive overexpressors. Additionally, *OsRAmy3D* and *OsLIC* which have been previously reported to be induced by BZR1 in rice (Qiao et al. 2017; Xiong et al. 2022) were significantly upregulated in the constitutive overexpressor (Fig. 5F; Supplementary Data Set 4). Transcripts from 2 BR biosynthesis genes *OsDWF4* and *OsDWF2* were less abundant when *OsBZR1* was expressed constitutively and when expressed in the BS (Fig. 5F; Supplementary Data Set 4). Additionally, 2 genes associated with BR catabolism *OsCYP734A2* and *OsCYP734A4* were significantly upregulated but only in the ubiquitous overexpressors (Fig. 5F; Supplementary Data Set 4).

Finally, we interrogated the data to better understand how *OsBZR1* overexpression impacted genes involved in chloroplast function and biogenesis. We detected relatively few changes here, possibly due to the fact that the transcriptome of mature leaves was analyzed at a stage during which chloroplast development may be complete. Transcripts derived from the chlorophyll biosynthesis gene *OsPORA* were more abundant in ubiquitous overexpressors, but this was not the case when *OsBZR1* was expressed in the BS (Fig. 5F; Supplementary Data Set 4). The *OsGLK2* transcription factor was significantly downregulated in one of the ubiquitous overexpressing lines but slightly upregulated when *OsBZR1* was overexpressed in the BS (Fig. 5F; Supplementary Data Set 4). Two genes involved in chloroplast division *PDV1a* and *PDV1c* were decreased when *OsBZR1* was constitutively overexpressed which could indicate the mechanism behind BZR1-modulated changes in chloroplast area (Fig. 5F; Supplementary Data Set 4). The expression of other master regulators of chloroplast development (*OsCGA1*, *OsGNC*, *OsMYBS1*, and *MYB-related family protein*) was unchanged when *OsBZR1* was overexpressed (Supplementary Fig. S14).

## Discussion

### A role for BRs in controlling chloroplast size and number

As key phytohormones, BRs have been studied for decades with much of the work performed in Arabidopsis. One particularly well-documented role is the control of photomorphogenesis, including the induction of hypocotyl elongation combined with the inhibition of cotyledon expansion, chlorophyll biosynthesis, and chloroplast differentiation in the dark (Chory et al. 1991; Asami et al. 2000; Komatsu et al. 2010; Yu et al. 2011; Zhang et al. 2021; Tachibana et al. 2022). BRs are important for chloroplast development in Arabidopsis by modulating the activity of GOLDEN2-LIKE transcription factors (GLK1&2), master regulators of chloroplast development which control the expression of chloroplast targeted proteins encoded by photosynthesis-associated nuclear genes (*PhANGs*) important for the correct development of chloroplast ultrastructure and chlorophyll accumulation (Zhang et al. 2021; Tachibana et al. 2024). However, to our knowledge, there are no reports demonstrating that BRs control chloroplast size or number in Arabidopsis or rice. The results reported here show that exogenous treatment with an active BR and a BR biosynthesis inhibitor altered chloroplast planar area and number in rice BS cells and both ubiquitous and cell-specific overexpression of *OsBZR1* resulted in increased chloroplast area. The transcript abundance of *OsPDV1a* and *OsPDV1c*, genes involved in chloroplast division, was significantly decreased upon overexpression of *OsBZR1*. However, the overexpression lines showed no biologically meaningful changes in BS cell chloroplast number. These results imply that overexpression of BZR1 does not have a material effect on chloroplast division (despite the fact that the expression of PDVs was downregulated in the UBQ OE lines). Together, these results support a previously unknown role for BRs in modulating chloroplast size throughout plant development.

### Using OsBZR1 to engineer chloroplast volume specifically in rice BS cells

The role of BRs in increasing chloroplast area provided a candidate to manipulate the chloroplast compartment of rice BS cells, an important characteristic if C_4_ photosynthesis is to be engineered into this species (Hibberd et al. 2008). Although ubiquitous overexpression of *OsBZR1* resulted in a significant increase in the area of individual chloroplasts in the BS, it did not impact M chloroplast area. This is possibly because the chloroplast compartment of M cells is already large, and so there is limited capacity to increase this further. However, this is not consistent with the BS cell-specific overexpressor where 2 of the 3 lines showed significant increases in the area of individual M chloroplasts. Therefore, it is possible that other factors limit chloroplast area in the M cells of the ubiquitous overexpressor and further analysis, for example, use of single cell transcriptomics, would be valuable to elucidate these mechanisms.

Although the increased BS cell chloroplast area in the UBQ OE plants was extremely promising in terms of engineering this cell type, it had severe effects on plant health and yield. This is consistent with previous reports of misexpression of components of BR biosynthesis or signaling leading to defects in plant growth (Clouse et al. 1996; Kim et al. 2020; Nolan et al. 2020; Manghwar et al. 2022). Exogenous application of BRs to Arabidopsis has been shown to have growth-promoting effects when a low dose is applied, while higher doses show growth retardation (González-García et al. 2011; Chaiwanon and Wang 2015; Nolan et al. 2020). The growth defects reported here for rice demonstrate conservation in the BR signaling pathway between rice and Arabidopsis. Transcriptome analyses of the *OsBZR1* ubiquitous overexpressors showed differentially expressed genes (DEGs) with enriched GO terms including “biotic stress response”, “abiotic stress response”, and “senescence”, consistent with the negative effects on plant development.

Endogenous tissue-specific control of BR signaling ensures proper growth and avoids deleterious effects of ubiquitous BR action (Nolan et al. 2020). Indeed, studies using tissue-specific promoters to complement BR mutant phenotypes have revealed that cell type-specific confinement of BR signaling is essential for proper shoot and root development (Chaiwanon and Wang 2015; Hacham et al. 2011; Kang et al. 2017; Nolan et al. 2020, 2023; Savaldi-Goldstein et al. 2007; Vragović et al. 2015). This cell type-specific signaling can be harnessed for the development of plants with improved stress resistance and yield. For example, overexpression of BRL3, a vascular-enriched BR receptor in Arabidopsis, conferred drought stress tolerance without a growth penalty whereas altering the ubiquitously expressed BRI1 receptor conferred drought tolerance but at the expense of growth (Fàbregas et al. 2018). And overexpression of C-22 hydroxylases, which increase BR levels, using a promoter that limits expression mainly to vegetative organs resulted in significant increases in grain yield (Wu et al. 2008). The results reported here therefore confirm that endogenous tissue-specific control of BR signaling is important in rice.

As cell-specific gene expression in Arabidopsis and rice had proved useful (Fàbregas et al. 2018), we used a BS cell-specific promoter for rice (Hua et al. 2024) to express *OsBZR1* only in BS cells. This resulted in an increase in BS cell chloroplast area of up to 34% and had no detectable negative impacts on plant growth. Moreover, the transcriptome of these plants showed no enrichment in stress- or senescence-related GO terms, indicating that the cell-specific overexpression was successful in avoiding off-target perturbations. In fact, the transcriptome of these plants showed very little change compared with the corresponding nulls. A contributing factor to this outcome is likely that BS only makes up approximately 15% of chloroplast-containing leaf cells (Leegood 2008). It was also noticeable that these lines showed little to no change in net photosynthesis or seed yield, again possibly due to the small proportion of BS cells in the leaf.

### Exploiting multiple master regulators to manipulate chloroplast development

The work presented here investigated the potential of manipulating BR signaling to increase the chloroplast compartment in rice BS cells to mimic a more C_4_-like leaf anatomy. The percentage increases in BS chloroplast area of the ubiquitous and cell-specific *OsBZR1* overexpression lines reported here are comparable with other work describing master regulators of chloroplast development in rice. For example, overexpression of *ZmGLK* resulted in increases in chloroplast area of approximately 30% (Wang et al. 2017) and BS cell-specific overexpression of *OsCGA1* resulted in a 3.5-fold increase in chloroplast size and a significant increase in proportion of the BS cell occupied by chloroplasts (Lee et al. 2021). Based on these similarities in changes in chloroplast size, BZR1 could be considered as a promising candidate for engineering the chloroplast compartment in rice. In the controlled conditions we used, we observed no changes to photosynthesis or yield when *OsBZR1* was overexpressed, which contrasts with overexpression of *ZmGLK* that resulted in a 30 to 40% increase in vegetative biomass and grain yield in the field (Li et al. 2020). It may therefore be interesting to assess these *OsBZR1* overexpression lines in the field. In contrast to Arabidopsis, where BZR1 has been shown to modulate *GLK* expression (Yu et al. 2011), our transcriptome data showed no consistent changes to *OsGLK* when *OsBZR1* was overexpressed. Additionally, the expression of other master regulators of chloroplast development (including *OsCGA1*, *OsGNC*, and *OsMYBS* genes putatively orthologous to MYB-related transcription factors that were recently shown to control chloroplast biogenesis in *Marchantia polymorpha* and Arabidopsis) were unchanged when *OsBZR1* was overexpressed (Supplementary Fig. S14). Thus, one could consider combining these regulators to test for additive or synergistic effects. To conclude, although work is still needed to elucidate precisely how BRs and BZR1 manipulate chloroplast size and number, the results shown here present a promising candidate to be harnessed to manipulate chloroplast development in rice and, potentially, other important crop species.

## Materials and methods

### Plant material and growth conditions

For seed propagation and phenotyping experiments, seeds of wild-type rice (*O. sativa* spp. *japonica* cv. Kitaake) and transgenic rice lines (UBQ Null, UBQ OE, BS Null, and BS OE) were imbibed in sterile Milli-Q water and incubated at 28 °C in the dark for 2 d. Seeds were transferred to Petri plates with moistened Whatman filter paper and germinated in the growth cabinet at 28 °C with a 16/8 h light/dark cycle for a further 2 d. Germinated seedlings were placed into 9 × 9 cm pots (2 plants per pot) filled with Profile Field and Fairway soil amendment (www.rigbytaylor.com) and grown in a walk-in plant growth chamber under a 12 h photoperiod at a photon flux density of 400 *μ*mol m^−2^ s^−1^ at 28 °C day and 20 °C night. Plants were fed once a week with Peters Excel Cal-Mag Grower fertilizer solution (LBS Horticulture, Clone, UK) at a concentration of 0.33 g/L with additional iron (Fe7 EDDHA regular, Gardening Direct, UK) at a concentration of 0.065 g/L. Once fully expanded, Leaf 4 and/or Leaf 8 were harvested for phenotyping.

### Production of transgenic rice lines

All constructs were generated using the Golden Gate cloning system, with *Bpi*I and *Bsa*I enzymes used to create Level 1 and Level 2 modules, respectively (Engler et al. 2009; Weber et al. 2011). The full-length cDNA sequence of *OsBZR1* (LOC_Os07g39220/Os07g0580500) was rice codon optimized for ease of detection against the endogenous *OsBZR1* gene and domesticated to remove internal *Bpi*I and *Bsa*I restriction sites (Supplementary Data Set 5). This sequence was then synthesized for cloning. For ubiquitous *OsBZR1* overexpression, the rice codon-optimized *OsBZR1* sequence was cloned downstream of the maize (*Z. mays*) *UBIQUITIN* promoter (p*ZmUBI*) and upstream of a nos terminator (nost) (Supplementary Fig. S6A). The p*ZmUBI* was made up of 983 bp upstream of the transcription start site and 1,014 bp of the first intron (Supplementary Data Set 5). For the BS cell-specific *OsBZR1* line, the rice codon-optimized *OsBZR1* sequence was cloned downstream of the rice *SULFITE REDUCTASE* promoter (p*OsSIR*) (Hua et al. 2024) and upstream of a nos terminator (nost) (Supplementary Fig. S10A). The Level 1 Golden Gate modules were confirmed through sequencing using backbone specific primers: pL1-F: GCGGACGTTTTTAATGTACTG and pL1-R: CCAATATATCCTGTCAAACACTG. Confirmed Level 1 constructs were then cloned with a hygromycin resistance Level 1 module which contained the hygromycin resistance gene (Supplementary Data Set 5) downstream of the rice *ACTIN* promoter (p*OsACT)* (Supplementary Data Set 5) and upstream of a nos terminator. This module was used for selection of transformants at T_0_ stage and selection of homozygous lines at T_2_ stage.

For the *OsBZR1* RNAi lines, the native *OsBZR1* coding sequence was submitted to WeigelWorld WMD amiRNA design website 3.1.18 (http://wmd3.weigelworld.org/), designing against the rice cDNA dataset (v6.1 MLU). The top 2 microRNA sequences were selected and cloned, along with specific stem loop sequences, downstream of the maize *UBIQUITIN* promoter (*pZmUBI*). These Level 1 modules were then cloned with a hygromycin resistance Level 1 module (p*OsACT-HYG*) and upstream of a nos terminator (Supplementary Fig. S5A).

MicroRNA sequences used:

*bzr1-1:* TTTAACGGTACGTCACAGCGA
*bzr1-2:* TACAAGATTAACCTAAGGCTG

Once cloning of each construct was complete, *O. sativa* spp. *japonica* cv. Kitaake was transformed using *Agrobacterium tumefaciens* as described previously (Hiei and Komari 2008) with several modifications. Seeds were de-husked and sterilized with 10% (v/v) bleach for 15 min before placing them on nutrient broth (NB) callus induction media containing 2 mg/L 2,4-dichlorophenoxyacetic acid for 4 wk in the dark at 28 °C. Calli were co-incubated with *A. tumefaciens* strain LBA4404 carrying the expression plasmid of interest in NB inoculation medium containing 40 *μ*g/mL acetosyringone for 3 d in the dark at 22 °C. Calli were transferred to NB recovery medium containing 300 mg/L timentin for 1 wk in the dark at 28 °C. They were then transferred to NB selection medium containing 35 mg/L hygromycin B for 4 wk in the dark at 28 °C. Proliferating calli were subsequently transferred to NB regeneration medium containing 100 mg/L myo-inositol, 2 mg/L kinetin, 0.2 mg/L 1-naphthaleneacetic acid, and 0.8 mg/L 6-benzylaminopurine for 4 wk in the light at 28 °C. Plantlets were transferred to NB rooting medium containing 0.1 mg/L 1-naphthaleneacetic acid and incubated in magenta pots for 2 wk in the light at 28 °C. Finally, plants were transferred into Profile Field and Fairway soil amendment (www.rigbytaylor.com) and grown in a walk-in plant growth chamber under a 12 h photoperiod at a photon flux density of 400 *μ*mol m^−2^ s^−1^ at 28 °C day and 20 °C night. DNA was isolated from individual T_0_ plants and DNA blot analysis performed to determine insertion copy number. Lines with single insertions in different locations of the genome were used for phenotyping experiments.

### BL and Brz treatments and chlorophyll analysis

*O. sativa* spp. *japonica* cv. Kitaake seeds were de-husked and sterilized in 10% (v/v) bleach for 30 min. After washing several times with sterile water, seeds were imbibed in water and incubated at 28 °C in the dark for 2 d. For de-etiolation experiments, seedlings germinated in the dark were transferred in a dark room equipped with a green light into ½ strength Murashige and Skoog (MS) medium (0.8% agar) supplemented with either 10 *µ*M BL (Santa Cruz Biotechnology, Inc.) or 10 *µ*M Brz (Merck Life Science UK Ltd., Gillingham, UK). Magentas were covered in aluminum foil and placed in a growth cabinet set to a 28 °C, 16 h day and 20 °C, 8 h night cycle for a further 3 d. At the beginning of the fourth photoperiod, aluminum foil was removed from the magentas to expose the seedlings to light. Leaf tissue was harvested for chlorophyll quantification and confocal microscopy at 0, 4, 12, and 24 h after exposure to light. For later stages, sterile germinated seedlings were transferred into ½ strength MS medium (0.8% agar) supplemented with either 10 *µ*M BL or 10 *µ*M Brz and placed in a growth cabinet set to a 28 °C, 16 h day and 20 °C, 8 h night cycle until Leaf 4 had fully expanded, at which time it was harvested for chlorophyll quantification and confocal microscopy. The concentration of BL was selected after performing the de-etiolation and later time point experiments using a concentration gradient, including 0, 2, 5, and 10 *µ*M. The 10 *µ*M concentration had the greatest and most consistent impact on plant morphology, BS chloroplast area, and the number of chloroplasts per BS cell (Supplementary Fig. S1) and was thus used for downstream experiments. This concentration has also been used previously in rice (Bai et al. 2007).

Tissue for chlorophyll quantification (Leaf 4, Leaf 8, or seedlings during de-etiolation experiments) was harvested, weighed, and immediately flash-frozen in liquid nitrogen. Frozen tissue was ground into a fine powder and suspended in 1 mL of 80% (v/v) acetone. After vortexing, the tissue was incubated on ice for 15 min with occasional mixing of the suspension. The tissue was spun at 13,000 rpm for 5 min at 4 °C and supernatant removed. The extraction was repeated and supernatants pooled before measuring absorbance at 663.6 and 646.6 nm. Total chlorophyll content was determined as described previously (Porra et al. 1989).

### Chloroplast imaging and quantification

Chloroplasts in individual BS and M cells were imaged and quantified using light and confocal laser microscopy. To isolate single M and BS cells for light microscopy, cells from Leaf 8 of wild-type Kitaake or transgenic rice lines (UBQ Null, UBQ OE, BS Null, and BS OE) were isolated following the protocol of Khoshravesh and Sage (2018). Briefly, the middle region of fully expanded Leaf 8 was cut into 5 mm-long × 2 mm-wide strips along the leaf proximodistal axis using a razorblade and immediately immersed in room temperature 4% w/v paraformaldehyde (pH 6.9) (Thermo Fisher Scientific Inc.). Fixed tissue was left in paraformaldehyde at 4 °C for at least 1 h (up to overnight). Cell walls were then digested by incubating in 0.2 m sodium-EDTA (pH 9.0) at 55 °C for 2 h, rinsed in digestion buffer (0.15 m Na_2_HPO_4_, 0.04 m citric acid, pH 5.3) and then incubated in 2% w/v pectinase from *Aspergillus niger* (Merck Life Science UK Ltd., Gillingham, UK) in digestion buffer at 45 °C for 2 h. Digestion was stopped by incubation in empty digestion buffer twice for 30 min at room temperature. After digestion, individual cells were released by mechanical disruption using the bottom of an Eppendorf tube. Isolated M and BS cells were imaged by brightfield microscopy using an Olympus BX41 microscope (Olympus UK and Ireland, Southend-on-Sea, UK), recording each cell in the paradermal plane where most of the chloroplasts were in focus. Images were captured using an MP3.3-RTV-R-CLR-10-C MicroPublisher camera and QCapture Pro 7 software (Teledyne Photometrics, Birmingham, UK). The area of individual M and BS cell chloroplasts in each image was quantified using ImageJ version 1.53k.

To visualize and quantify chloroplasts in BS cells using a confocal laser microscope, the methods described in Billakurthi and Hibberd (2023) were followed. Briefly, the middle region of fully expanded Leaf 4 and Leaf 8 was fixed with 1% (w/v) glutaraldehyde (Thermo Fisher Scientific Inc.) in 1× PBS buffer. Samples were left in fixative for 2 h and then washed twice with 1× PBS buffer. Before confocal microscopy, the adaxial side of the fixed leaf material was ablated gently with a fine razor blade to remove the cuticle and M layers and then incubated in calcofluor white (0.1%; Sigma) for 5 min to stain cell walls prior to rinsing twice with H_2_O. A Leica SP8X confocal microscope upright system (Leica Microsystems) was used for fluorescence imaging of the BS cell chloroplasts and cell walls. The microscope has 2 continuous wave laser lines, 405 and 442 nm, a 460 to 670 nm super continuum white light laser (WLL), 4 hybrid detectors, and 1 photomultiplier tube. Imaging was conducted using a 25× water immersion objective and Leica Application Suite X software (LAS X; version: 3.5.7.23225). Calcofluor white was excited at 405 nm and emitted fluorescence captured from 452 to 472 nm, and gain was adjusted for each image. Chlorophyll autofluorescence was excited at 488 nm and emission captured 672 to 692 nm, and gain was set at 100 for all images. For all lines, Leaf 4 and Leaf 8 from 4 plants were assessed, with 3 different intermediate veins imaged in each leaf. The planar area of individual M and BS chloroplasts in each image was quantified using ImageJ version 1.53k.

### Serial block-face SEM

To visualize the ultrastructure of individual chloroplasts, serial block-face SEM was used. For this, the middle region of fully expanded Leaf 8 was cut into 2 × 2 mm squares and fixed in 2% (v/v) glutaraldehyde and 2% (w/v) formaldehyde in 0.05 to 0.1 m sodium cacodylate (NaCac) buffer (pH 7.4) containing 2 mm calcium chloride. Samples were vacuum infiltrated overnight, washed 5 times in 0.05 to 0.1 m NaCac 557 buffer, and post-fixed in 1% (v/v) aqueous osmium tetroxide, 1.5% (w/v) potassium ferricyanide in 0.05 m NaCac buffer for 3 d at 4 °C. After osmication, samples were washed 5 times in deionized water and post-fixed in 0.1% (w/v) thiocarbohydrazide for 20 min at room temperature in the dark. Samples were then washed 5 times in deionized water and osmicated for a second time for 1 h in 2% (v/v) aqueous osmium tetroxide at room temperature. Samples were washed 5 times in deionized water and subsequently stained in 2% (w/v) uranyl acetate in 0.05 m maleate buffer (pH 5.5) for 3 d at 4 °C and washed 5 times afterwards in deionized water. Samples were then dehydrated in an ethanol series and transferred to acetone and then to acetonitrile. Leaf samples were embedded in Quetol 651 resin mix (TAAB Laboratories Equipment Ltd.) and cured at 60 °C for 2 d. Ultra-thin sections of embedded leaf samples were prepared and placed on Melinex (TAAB Laboratories Equipment Ltd.) plastic coverslips mounted on aluminum SEM stubs using conductive carbon tabs (TAAB Laboratories Equipment Ltd.), sputter-coated with a thin layer of carbon (∼30 nm) to avoid charging and imaged in a Verios 460 SEM at 4 keV accelerating voltage and 0.2 nA probe current using the concentric backscatter detector in field free (low magnification) or immersion (high magnification) mode (working distance 3.5 to 4 mm, dwell time 3 *μ*s, 1,536 ×1,024 pixel resolution). SEM-stitched maps were acquired at 10,000 × magnification using the FEI MAPS automated acquisition software. Grayscale contrast of the images was inverted to allow easier visualization.

### Gas exchange measurements

Fully expanded Leaf 8 was used to measure photosynthetic rates using a LI-6800 photosynthesis system (LICOR Biosciences). For the UBQ OE plants, measurements were performed prior to the onset of early senescence. Four individual plants were sampled per line. Measurements were made at a constant airflow of 400 *μ*mol s^−1^, leaf temperature of 30 °C, and relativity humidity of 60%. Leaves were acclimated in the chamber for approximately 10 min before net photosynthesis (CO_2_ assimilation rate—A) measurements were made at ambient conditions (light intensity of 400 *μ*mol photons m^−2^ s^−1^ and intercellular CO_2_ concentration (Ci) of 400 *μ*mol CO_2_ mol^−1^ air). All measurements were performed on the midportion of the leaf blade.

### Phylogenetic tree inference

To identify putative rice orthologs of transcription factors that mediate BR-responsive gene expression in Arabidopsis (*A. thaliana*), the protein-coding genes derived from representative gene models were downloaded from Phytozome (Goodstein et al. 2012). These proteomes were subject to orthogroup inference using OrthoFinder (Emms and Kelly 2015, 2019). The orthogroup containing Arabidopsis BZR1, BES1, (BZR2), BEH1, BEH2, BEH3, and BEH4 was identified. The sequences from this orthogroup were subject to multiple sequence alignment using MergeAlign (Collingridge and Kelly 2012) followed by bootstrapped maximum likelihood phylogenetic tree inference using IQTREE2 (Minh et al. 2020) with the best fitting model of sequence evolution (JTT + I + G4) inferred from the data.

### Total RNA extraction, cDNA library preparation, and transcriptome analysis

Total RNA was extracted from Leaf 4 using the RNeasy Plant Mini Kit (Qiagen, Germany) according to the manufacturer's instructions. Genomic DNA was removed from each sample using the RNase-Free DNase Set (Qiagen, Germany). RNA degradation and contamination were checked on 1% (w/v) agarose gels and then RNA quality and concentration determined with the RNA600 Pico Assay using the Agilent 2100 Bioanalyzer (Agilent Technologies, USA) and ND-200 NanoDrop (NanoDrop Technologies Inc., USA). RNA was sent to the Novogene Genomic Sequencing Centre (Cambridge) for library preparation and sequencing using the Illumina NovaSeq 600 PE150 sequencing platform and strategy. At least 6 Gb of raw data per sample was generated for subsequent transcriptome analysis. All transcriptome data has been deposited on NCBI in fasq format (PRJNA1234113 and PRJNA1234517).

Quality of raw sequencing data was assessed and controlled using the FastQC platform version 0.11.4 (Andrews 2010). Adapter trimming and filtering of all low-quality reads was performed using BBDuk (https://www.geneious.com/plugins/bbduk/) with the following parameters: k = 13, ktrim = r, useshortkmers = t, mink = 5, qtrim = r, trimq = 2- minlength = 50, ftl = 10, and ftr = 139. The *O. sativa* IRGSP-1.0 transcriptome was downloaded from Ensemble Plants (https://plants.ensembl.org/index.html) and used to build a Salmon reference index which was subsequently used to quantify the cleaned reads (Salmon version 1.5.2). For Salmon quantification (Patro et al. 2017), all parameters were left as default. The Salmon alignment and quantification results were checked using MultiQC (Ewels et al. 2016). To check that biological replicates clustered together and to visualize how the overexpression lines differed from corresponding null lines and from each other, a PCA was performed using the ggfortify package in R. Transcripts per million (TPM) counts from Salmon alignments were filtered for genes with at least 48 counts across all samples. The filtered data were then normalized to account for library size and transformed to a log scale using rlog transformation to allow for easier visualization. For the Pearson's correlation heatmap, the filtered, rlog transformed TPM data was used.

To determine changes in expression of individual genes between OE lines and corresponding null lines, the DESeq2 package (version 4.2) in R was used (Love Huber and Anders 2014). The quant.sf file generated for each sample from the Salmon quantification was used as the input for the DESeq2 analysis. Independent DESeq2 analyses were performed for the 2 different overexpression lines (i.e. UBQ OE samples versus corresponding UBQ Null samples and BSC OE samples versus corresponding BSC Null samples). Lists of significantly DEGs for further investigation were developed by filtering for genes with an adjusted *P* < 0.05. To gain further biological insight into DEG lists, GO enrichment analyses were performed using AgriGO v2 (Tian et al. 2017) with the *O. sativa* MSU7.0 genome set as the background reference.

### RT-qPCR

To check transgene expression levels, RNA was extracted from fully expanded Leaf 4 as described above. The total RNA was used as a template to synthesize cDNA using the SuperScript II Reverse Transcriptase kit (Invitrogen) according to the manufacturer's instructions. RT-qPCR was carried out on the cDNA using SYBR Green JumpStart Taq ReadyMix (Merck Life Science UK Ltd., Gillingham, UK) on a CFX384 Real-Time System (Bio-Rad) with the following cycle parameters; 94 °C for 2 min, (94 °C for 15 s, 60 °C for 1 min) × 40. This was performed using primers specific to the endogenous *OsBZR1* sequence (*endOsBZR1*) or primers specific to the rice codon-optimized *OsBZR1* sequence (*rcoOsBZR1*) and *OsEF-1*α and *OsUBQ* reference genes (Jain et al. 2006; Jain et al. 2018) with the following sequences:

*endOsBZR1*-F: ATGCTGCGATTTGGGCGATTTC

*endOsBZR1*-R: ACACAGAGATGAACAGTGAAGCC

*rcoOsBZR1-*F: CGTACAACCTCGTGAACCC

*rcoOsBZR1-*R: CGTCACCCTACCTTTGTCG

*OsEF-1*α*-*F: TTTCACTCTTGGTGTGAAGCAGAT

*OsEF-1*α*-*R: GACTTCCTTCACGATTTCATCGTAA

*OsUBI5*-F: ACCACTTCGACCGCCACTACT

*OsUBI5*-R: ACGCCTAAGCCTGCTGGTT

### Data analyses

Unless otherwise stated, all statistical analyses were performed using StatSoft Statistica software. Statistical analyses included 1-way ANOVA followed by Fisher Lsd post hoc analysis and independent *t*-tests. A *P* < 0.05 was considered significant. For instances where multiple hypothesis testing was performed (DEseq2 analyses), adjusted *P-*values (Benjamini–Hochberg) < 0.05 were considered significant. Refer to figure legends for specific statistical analyses conducted for each result presented.

### Accession numbers

Sequence data from this article can be found in the GenBank/EMBL data libraries under GeneIDs listed in Supplementary Data Set 4.

## Supplementary Material

kiaf108_Supplementary_Data

## Data Availability

The data underlying the transcriptome work presented in this article are available on NCBI (https://www.ncbi.nlm.nih.gov/) in fasq format, and can be accessed with PRJNA1234113 and PRJNA1234517.

## Supplementary data

The following materials are available in the online version of this article.

**Supplementary Figure S1.** Optimization of BL treatment concentration.

**Supplementary Figure S2.** BS cell area of untreated, BL-treated, and Brz-treated seedlings during de-etiolation.

**Supplementary Figure S3.** Number of veins in leaf 4 from untreated, BL-treated, and Brz-treated plants.

**Supplementary Figure S4.** Maximum likelihood phylogenetic tree of the *BZR1* gene family in plants.

**Supplementary Figure S5.** Development of *OsBZR1* knockout lines through RNAi.

**Supplementary Figure S6.** Development of rice lines constitutively overexpressing rice codon-optimized *OsBZR1* (UBQ OE).

**Supplementary Figure S7.** Constitutive overexpression of *OsBZR1* does not impact the number of chloroplasts per BS cell.

**Supplementary Figure S8.** Total chlorophyll content in Leaf 8 of UBQ Null and UBQ OE lines.

**Supplementary Figure S9.** BS cell size in UBQ Null and UBQ OE lines.

**Supplementary Figure S10.** Development of rice lines overexpressing rco*OsBZR1* specifically in BS cells (BS OE).

**Supplementary Figure S11.** BS cell-specific overexpression of *OsBZR1* does not impact chloroplast number per BS cell.

**Supplementary Figure S12.** BS cell-specific overexpression of *OsBZR1* does not impact BS cell size.

**Supplementary Figure S13.** Brz suppresses the positive effect of BS cell-specific overexpression of *OsBZR1*.

**Supplementary Figure S14.** Expression of other master regulators of chloroplast development in the constitutive and BS cell-specific *OsBZR1* overexpressors.

**Supplementary Data Set 1.** Significantly upregulated genes in each OsBZR1 overexpression line.

**Supplementary Data Set 2.** Significantly downregulated genes in each OsBZR1 overexpression line.

**Supplementary Data Set 3.** GO enrichment results of up- and downregulated DEGs from the UBQ and BSC OE lines.

**Supplementary Data Set 4.** DESEq2 results for genes of interest significantly differentially expressed in UBQ and BSC OE lines.

**Supplementary Data Set 5.** Sequences used for cloning.

## Dive Curated Terms

The following phenotypic, genotypic, and functional terms are of significance to the work described in this paper:

BZR1 Gramene: AT1G75080
BZR1 Araport: AT1G75080

BRL3 Gramene: AT3G13380
BRL3 Araport: AT3G13380

LAS Gramene: AT1G55580
LAS Araport: AT1G55580

cga1 Gramene: AT4G26150
cga1 Araport: AT4G26150

GNC Gramene: AT5G56860
GNC Araport: AT5G56860

SIR Gramene: AT5G04590
SIR Araport: AT5G04590

BIN2 Gramene: AT4G18710
BIN2 Araport: AT4G18710

BRI1 Gramene: AT4G39400
BRI1 Araport: AT4G39400

BEH3 Gramene: AT4G18890
BEH3 Araport: AT4G18890

OsBRI1 Gramene: Os01g0718300
OsBRI1 Araport: Os01g0718300

OsBZR1 Gramene: LOC_Os07g39220
OsBZR1 Araport: LOC_Os07g39220
