# Supplementary material for: Increased chloroplast area in the rice bundle sheath through cell-specific perturbation of brassinosteroid signaling
Source: Plant Physiol. 2025 Apr 2;197(4):kiaf108. doi: 10.1093/plphys/kiaf108 (PMC11997305; doi:10.1093/plphys/kiaf108)
Supplement: kiaf108_Supplementary_Data [file kiaf108_supplementary_data.zip › Cackett et al_ Supp. figures.pdf]

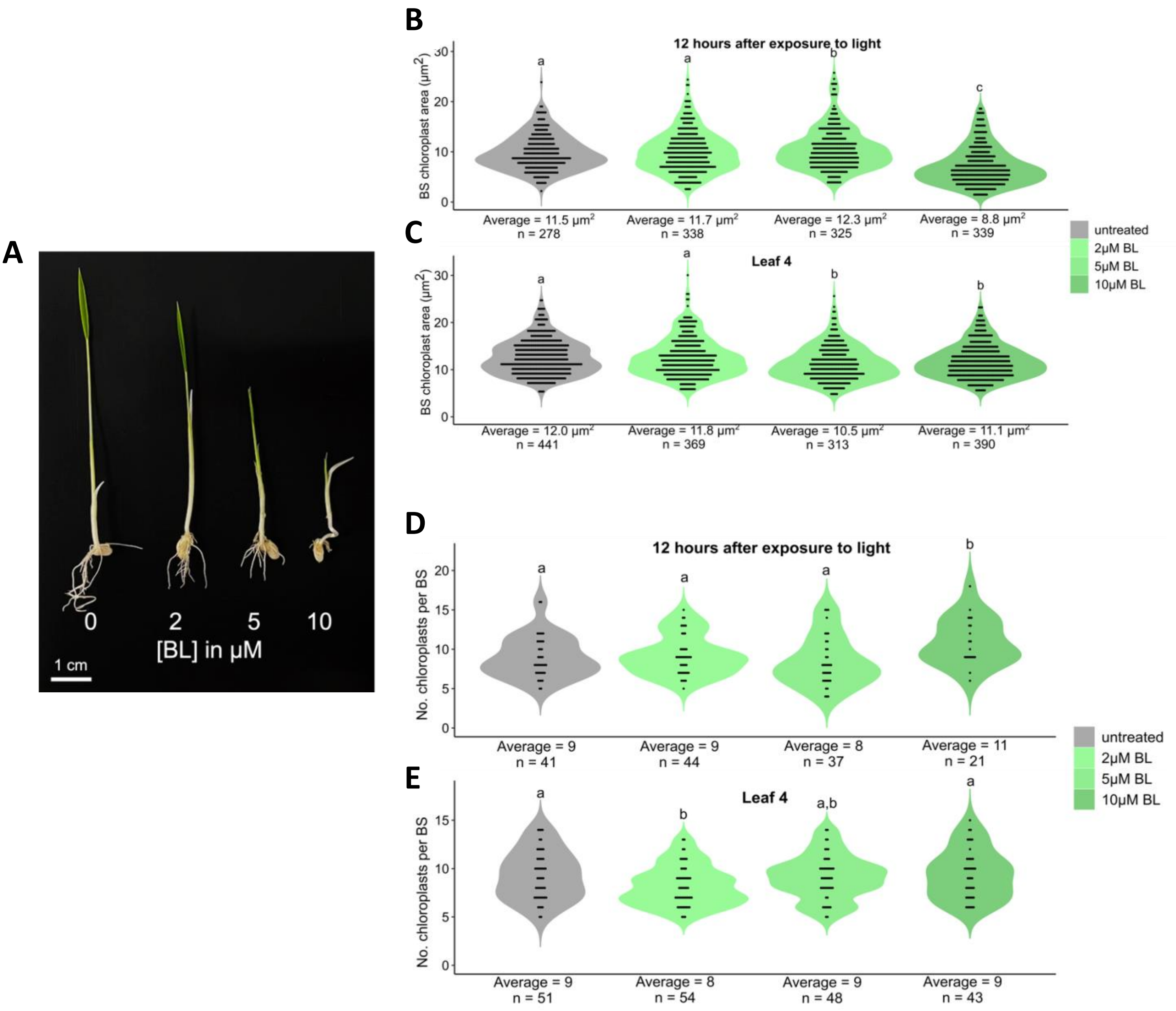

Supplemental Figure S1: Optimization of BL treatment concentration. To determine the optimal concentration of brassinolide (BL) for subsequent experiments, a BL concentration gradient was tested. Seeds were germinated in water and transferred to ½ MS-agar media with or without 2 µM, 5 µM or 10 µM BL. For the 12-hour time point, seedlings were transferred to light after 4 days in the dark and shoot tissue harvested 12 hours later for imaging using confocal laser scanning microscopy. For the leaf 4 time point, seedlings were transferred directly into light and fully expanded leaf 4 was harvested for imaging using confocal laser scanning microscopy. **A**: Representative images of seedlings from untreated, 2 µM, 5 µM and 10 µM BL treatment 12 hours after exposure to light. **B**: Bundle sheath cell chloroplast area in untreated, 2 µM, 5 µM and 10 µM BL treated seedlings 12 hours after exposure to light. **C**: Bundle sheath cell chloroplast area in leaf 4 from untreated, 2 µM, 5 µM and 10 µM BL treated seedlings. **D**: Number of chloroplasts per bundle sheath cell in untreated, 2 µM, 5 µM and 10 µM BL treated seedlings 12 hours after exposure to light. **E**: Number of chloroplasts per bundle sheath cell in leaf 4 from untreated, 2 µM, 5 µM and 10 µM BL treated seedlings. Data are

derived from confocal microscopy and from at least 250 chloroplasts and 20 cells for each treatment. For B, C, D and E Individual data points are overlaid as dots, where each dot represents a single observation. Dots are stacked symmetrically along the y-axis to visualize data density within each group. Letters above violins represent statistically significant differences ($p \leq 0.05$) in mean values as determined by Fisher LSD post-hoc analysis following a one-way ANOVA.

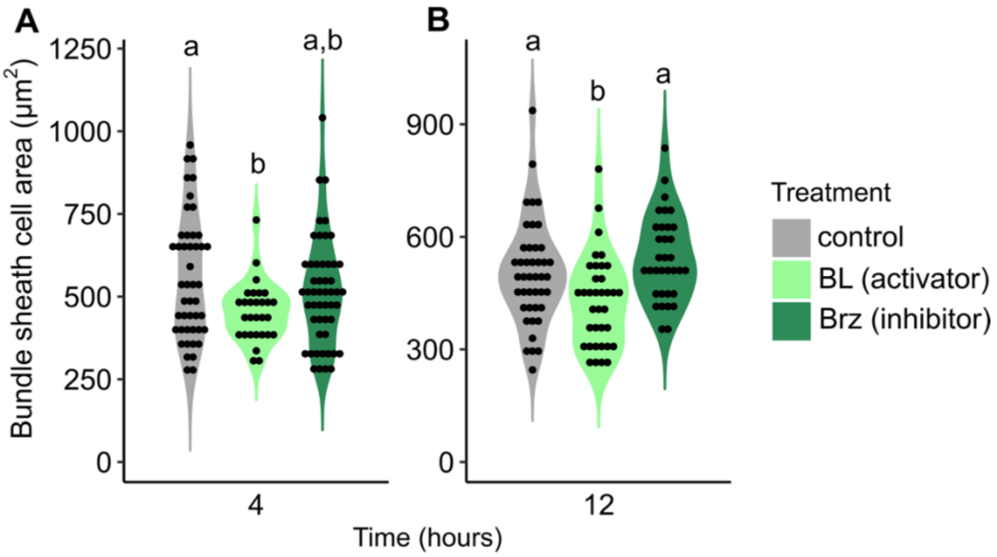

Supplemental Figure S2: Bundle sheath cell area of untreated, BL-treated and Brz-treated seedlings during de-etiolation. Seeds were germinated in water and transferred in the dark to ½ MS-agar media with or without 10 µM brassinolide (BL) or 10 µM brassinazole (Brz). After 4 days seedlings were transferred to light and shoot tissue harvested 0, 4, 12 and 24 hours later and fixed for imaging using confocal laser scanning microscopy. **A** and **B:** Bundle sheath cell area in control and BL/Brz treated seedlings 4 (**A**) and 12 (**B**) hours after exposure to light. Data are derived from confocal microscopy and from at least 30 cells for each timepoint in each treatment. For A and B Individual data points are overlaid as dots, where each dot represents a single observation. Dots are stacked symmetrically along the y-axis to visualize data density within each group. Letters above violins represent statistically significant differences ($p \leq 0.05$) in mean values as determined by Fisher LSD post-hoc analysis following a one-way ANOVA.

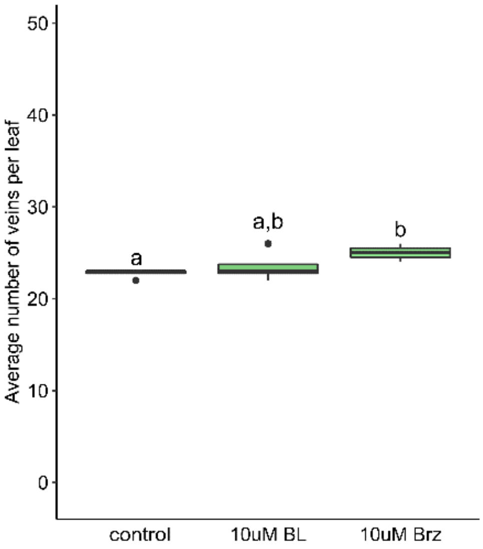

Supplemental Figure S3: Number of veins in leaf 4 from untreated, BL-treated and Brz-treated plants. Seeds were germinated in water and transferred to untreated MS media or MS media supplemented with 10 µM brassinolide (BL) or 10 µM brassinazole (Brz) and the number of veins in fully expanded leaf 4 counted. The box plots show the median and the interquartile range (IQR) between the first and third quartiles, whiskers indicate the smallest and largest values within 1.5 × IQR from the quartiles, while points beyond this range are considered outliers. Data are from 4 leaves for each treatment. Letters above boxes represent statistically significant differences ($p \leq 0.05$) in mean values as determined by Fisher LSD post-hoc analysis following a one-way ANOVA.

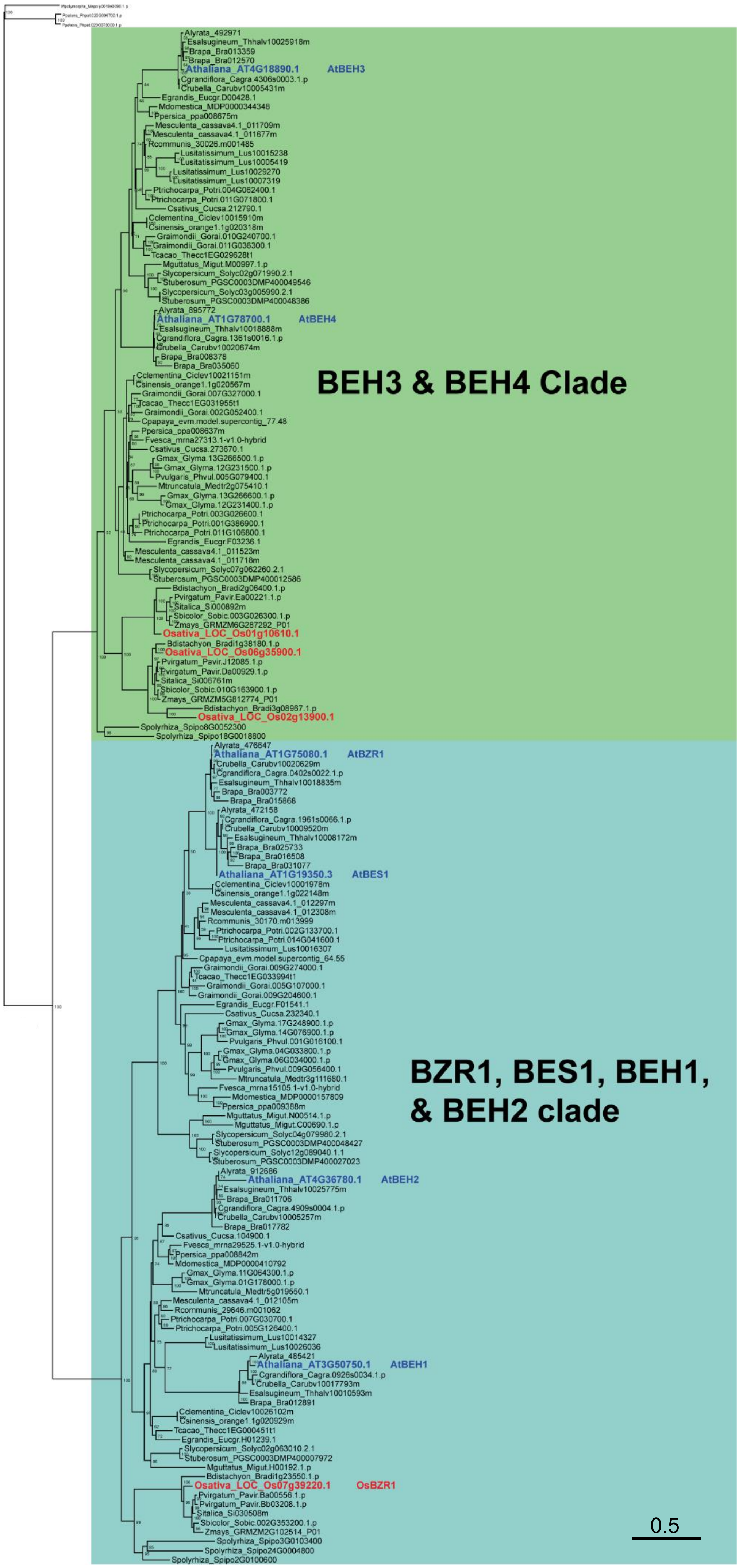

Alyrata_492971
Esalsugineum_Thhalv10025918m
Brapa_Bra013359
Brapa_Bra012570
Athaliana_AT4G18890.1
AtBEH3
Cgrandiflora_Cagra.4306s0003.1.p
Crubella_Carubv10005431m
Egrandis_Eucgr.D00428.1
Mdomestica_MDP0000344348
Ppersica_ppa008675m
Mesculenta_cassava4.1_011709m
Mesculenta_cassava4.1_011677m
Rcommunis_30026.m001485
Lusitatissimum_Lus10015238
Lusitatissimum_Lus10005419
Lusitatissimum_Lus10029270
Lusitatissimum_Lus10007319
Ptrichocarpa_Potri.004G062400.1
Ptrichocarpa_Potri.011G071800.1
Csativus_Cucsa.212790.1
Cclementina_Ciclev10015910m
Csinensis_orange1.1g020318m
Graimondii_Gorai.010G240700.1
Graimondii_Gorai.011G036300.1
Tcacao_Thecc1EG029628t1
Mguttatus_Migut.M00997.1.p
Slycopersicum_Solyc02g071990.2.1
Stuberosum_PGSC0003DMP400049546
Slycopersicum_Solyc03g005990.2.1
Stuberosum_PGSC0003DMP400048386
Alyrata_895772
Athaliana_AT1G78700.1
AtBEH4
Esalsugineum_Thhalv10018888m
Cgrandiflora_Cagra.1361s0016.1.p
Crubella_Carubv10020674m
Brapa_Bra008378
Brapa_Bra035060
BEH3 & BEH4 Clade
Cclementina_Ciclev10021151m
Csinensis_orange1.1g020567m
Graimondii_Gorai.007G327000.1
Tcacao_Thecc1EG031955t1
Graimondii_Gorai.002G052400.1
Cpapaya_evm.model.supercontig_77.48
Ppersica_ppa008637m
Fvesca_mrna27313.1-v1.0-hybrid
Csativus_Cucsa.273670.1
Gmax_Glyma.13G266500.1.p
Gmax_Glyma.12G231500.1.p
Pvulgaris_Phvul.005G079400.1
Mtruncatula_Medtr2g075410.1
Gmax_Glyma.13G266600.1.p
Gmax_Glyma.12G231400.1.p
Ptrichocarpa_Potri.003G026600.1
Ptrichocarpa_Potri.001G386900.1
Ptrichocarpa_Potri.011G106800.1
Egrandis_Eucgr.F03236.1
Mesculenta_cassava4.1_011523m
Mesculenta_cassava4.1_011718m
Slycopersicum_Solyc07g062260.2.1
Stuberosum_PGSC0003DMP400012586
Bdistachyon_Bradi2g06400.1.p
Pvirgatum_Pavir.Ea00221.1.p
Sitalica_Si000892m
Sbicolor_Sobic.003G026300.1.p
Zmays_GRMZM6G287292_P01
Osativa_LOC_Os01g10610.1
Bdistachyon_Bradi1g38180.1.p
Osativa_LOC_Os06g35900.1
Pvirgatum_Pavir.J12085.1.p
Pvirgatum_Pavir.Da00929.1.p
Sitalica_Si006761m
Sbicolor_Sobic.010G163900.1.p
Zmays_GRMZM5G812774_P01
Bdistachyon_Bradi3g08967.1.p
Osativa_LOC_Os02g13900.1
Spolyrhiza_Spipo8G0052300
Spolyrhiza_Spipo18G0018800
Alyrata_476647
Athaliana_AT1G75080.1
AtBZR1
Crubella_Carubv10020629m
Cgrandiflora_Cagra.0402s0022.1.p
Esalsugineum_Thhalv10018835m
Brapa_Bra003772
Brapa_Bra015868
Alyrata_472158
Cgrandiflora_Cagra.1961s0066.1.p
Crubella_Carubv10009520m
Esalsugineum_Thhalv10008172m
Brapa_Bra025733
Brapa_Bra016508
Brapa_Bra031077
Athaliana_AT1G19350.3
AtBES1
Cclementina_Ciclev10001978m
Csinensis_orange1.1g022148m
Mesculenta_cassava4.1_012297m
Mesculenta_cassava4.1_012308m
Rcommunis_30170.m013999
Ptrichocarpa_Potri.002G133700.1
Ptrichocarpa_Potri.014G041600.1
Lusitatissimum_Lus10016307
Cpapaya_evm.model.supercontig_64.55
Graimondii_Gorai.009G274000.1
Tcacao_Thecc1EG033994t1
Graimondii_Gorai.005G107000.1
Graimondii_Gorai.009G204600.1
Egrandis_Eucgr.F01541.1
Csativus_Cucsa.232340.1
Gmax_Glyma.17G248900.1.p
Gmax_Glyma.14G076900.1.p
Pvulgaris_Phvul.001G016100.1
Gmax_Glyma.04G033800.1.p
Gmax_Glyma.06G034000.1.p
Pvulgaris_Phvul.009G056400.1
Mtruncatula_Medtr3g111680.1
Fvesca_mrna15105.1-v1.0-hybrid
Mdomestica_MDP0000157809
Ppersica_ppa009388m
Mguttatus_Migut.N00514.1.p
Mguttatus_Migut.C00690.1.p
Slycopersicum_Solyc04g079980.2.1
Stuberosum_PGSC0003DMP400048427
Slycopersicum_Solyc12g089040.1.1
Stuberosum_PGSC0003DMP400027023
BZR1, BES1, BEH1, & BEH2 clade
Alyrata_912686
Athaliana_AT4G36780.1
AtBEH2
Esalsugineum_Thhalv10025775m
Brapa_Bra011706
Cgrandiflora_Cagra.4909s0004.1.p
Crubella_Carubv10005257m
Brapa_Bra017782
Csativus_Cucsa.104900.1
Fvesca_mrna29525.1-v1.0-hybrid
Ppersica_ppa008842m
Mdomestica_MDP0000410792
Gmax_Glyma.11G064300.1.p
Gmax_Glyma.01G178000.1.p
Mtruncatula_Medtr5g019550.1
Mesculenta_cassava4.1_012105m
Rcommunis_29646.m001062
Ptrichocarpa_Potri.007G030700.1
Ptrichocarpa_Potri.005G126400.1
Lusitatissimum_Lus10014327
Lusitatissimum_Lus10026036
Alyrata_485421
Athaliana_AT3G50750.1
AtBEH1
Cgrandiflora_Cagra.0926s0034.1.p
Crubella_Carubv10017793m
Esalsugineum_Thhalv10010593m
Brapa_Bra012891
Cclementina_Ciclev10026102m
Csinensis_orange1.1g020929m
Tcacao_Thecc1EG000451t1
Egrandis_Eucgr.H01239.1
Slycopersicum_Solyc02g063010.2.1
Stuberosum_PGSC0003DMP400007972
Mguttatus_Migut.H00192.1.p
Bdistachyon_Bradi1g23550.1.p
Osativa_LOC_Os07g39220.1
OsBZR1
Pvirgatum_Pavir.Ba00556.1.p
Pvirgatum_Pavir.Bb03208.1.p
Sitalica_Si030508m
Sbicolor_Sobic.002G353200.1.p
Zmays_GRMZM2G102514_P01
Spolyrhiza_Spipo3G0103400
Spolyrhiza_Spipo24G0004800
Spolyrhiza_Spipo2G0100600
0.5

Supplemental Figure S4. Maximum likelihood phylogenetic tree of the *BZR1* gene family in plants. *Arabidopsis thaliana* genes are highlighted in blue font with gene names added after the accession number. *Oryza sativa* accession numbers are highlighted in red font with *OsBZR1* indicated on the tree. Bootstrap support values shown at internal nodes. Scale bar indicates number of substitutions per aligned sequence site.

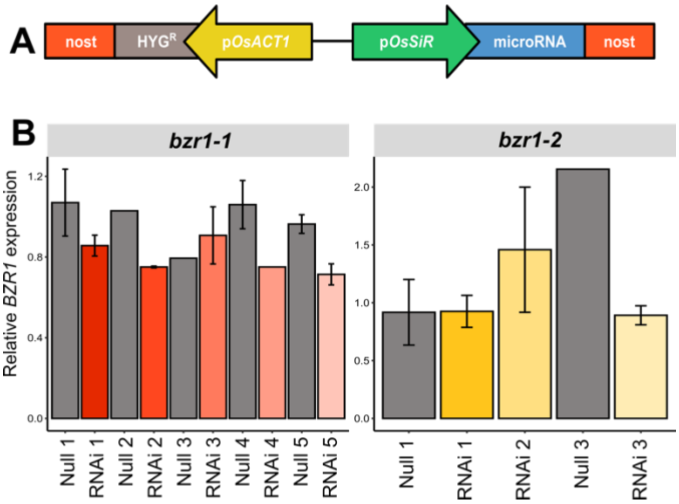

Supplemental Figure S5: Development of *OsBZR1* knockout lines through RNA interference (RNAi). A: Schematic of the construct used for transformation. The hygromycin resistance gene (HYG^R) driven by the ACTIN promoter (p*OsACT1*) was used to select transformants. The maize UBIQUITIN promoter (p*ZmUBI1*) was used to drive *OsBZR1* specific microRNA sequences for repression of expression. **B:** Homozygous lines in the T_2 generation were identified and the level of *OsBZR1* expression in leaf 4 determined by RT-qPCR using endogenous *OsBZR1* specific primers. Expression is shown relative to *OsUBI5* reference gene and represents the mean derived from one to four biological replicates. Error bars represent standard errors. The two different lines (*bzr1-1* and *bzr1-2*) were generated using different microRNA sequences.

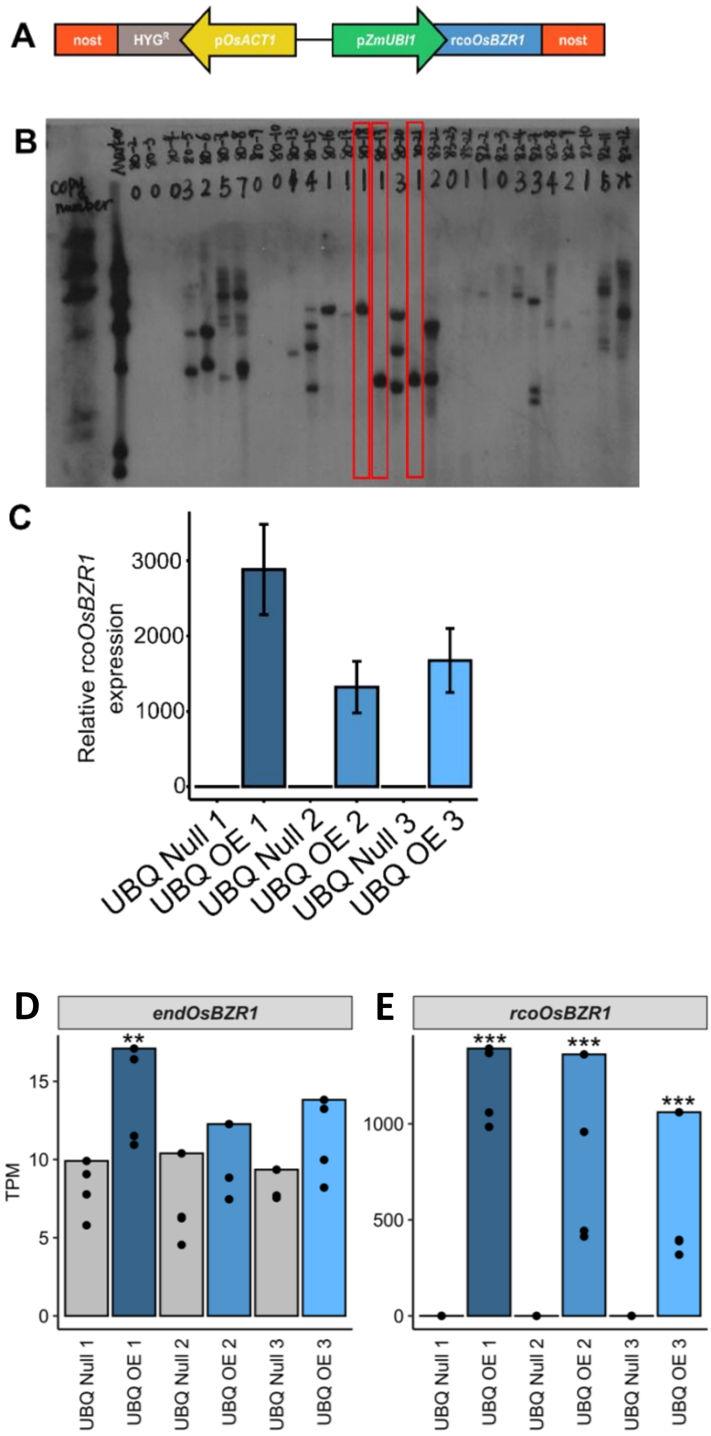

Supplemental Figure S6: Development of rice lines constitutively overexpressing rice codon-optimized OsBZR1 (UBQ OE). A: Schematic of the construct used for transformation. The hygromycin resistance gene (HYG^{R}) driven by the ACTIN promoter (p*OsACT1*) was used to select transformants. The maize UBIQUITIN promoter (p*ZmUBI1*) was used to drive constitutive expression of rice codon optimized *BZR1* (rco*OsBZR1*). **B:** Southern blot performed on DNA from T_0 transformants to identify lines containing single copies of the T-DNA insert. Red boxes outline the three independent, single copy lines chosen for phenotyping. 80-18, 80-19 and 80-21 refer to UBQ OE 1, UBQ OE 3 and UBQ OE 2 respectively **C:** Homozygous lines in the T_2 generation were identified and the level of rco*OsBZR1* expression in leaf 4 determined by RT-qPCR using rco*OsBZR1* specific primers.

Expression is shown relative to *OsEF1α* and *OsUBI5* reference genes and represents the mean derived from four biological replicates. Error bars represent standard errors. **D** and **E:** Endogenous (end*OsBZR1*) and rice codon optimized (rco*OsBZR1*) *OsBZR1* transcript abundance (transcripts per million – TPM) in homozygous T_2 plants was determined by bulk RNA sequencing**.** Data represents the mean derived from four biological replicates Stars above bars indicate a statistically significant difference between UBQ OE and corresponding null line as determined by independent t-test, where $p \leq 0.05$ is flagged with one star (*), $p \leq 0.01$ is flagged with 2 stars (**) and $p \leq 0.001$ is flagged with three stars (***).

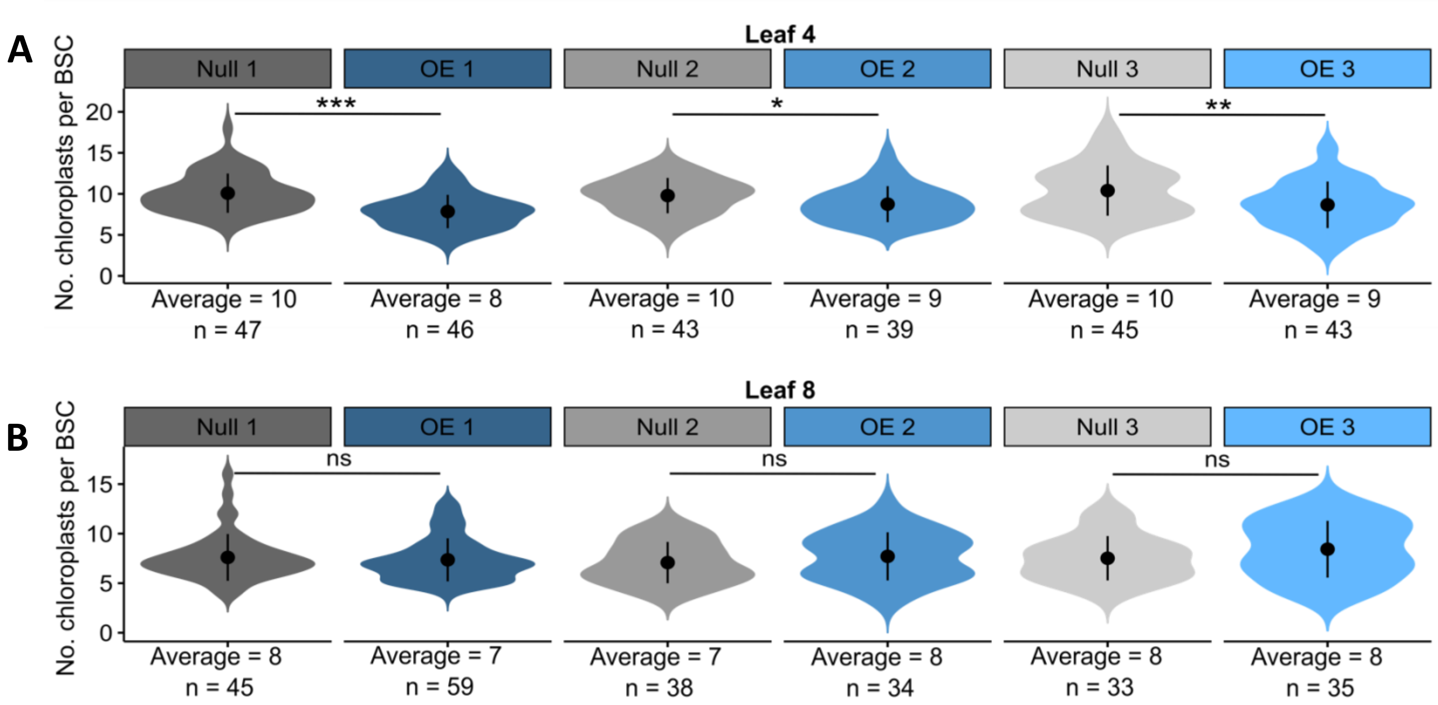

Supplemental Figure S7: Constitutive overexpression of *OsBZR1* does not impact the number of chloroplasts per bundle sheath cell. The rice codon optimized sequence for *BZR1* (rco*OsBZR1*) was cloned upstream of the maize *UBIQUITIN* promoter (p*ZmUBI*) and transformed into Kitaake rice to generate constitutive overexpression lines (referred to as UBQ OE). Confocal laser scanning microscopy was used to image bundle sheath cells (BSC) and chloroplasts in leaf 4 and leaf 8. **A** and **B:** The number of chloroplasts per bundle sheath cell in leaf 4 (**A**) and leaf 8 (**B**) was quantified from the confocal images. The center dot within each violin represents the mean number of chloroplasts per bundle sheath cell, while the vertical bars indicate ±1 standard deviation (SD) around the mean. Stars above violins indicate a statistically significant difference between overexpressor lines compared with corresponding null line as determined by independent t-test, where $p \leq 0.05$ is flagged with one star (*), $p \leq 0.01$ is flagged with 2 stars (**) and $p \leq 0.001$ is flagged with three stars (***). No statistically significant change is represented by "ns". The average below each violin is the mean number of chloroplasts per bundle sheath cell calculated for that line and n represents the number of BS cells assessed. Four biological replicates were used for each line.

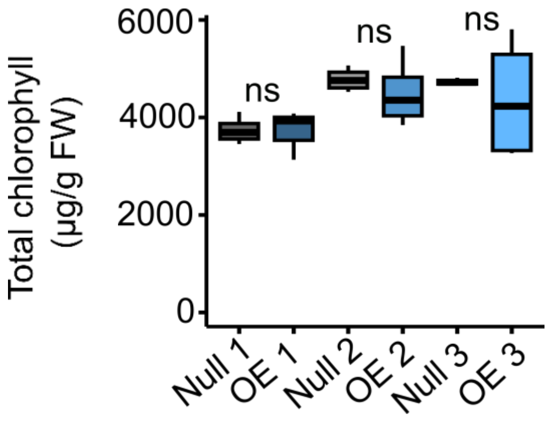

Supplemental Figure S8: Total chlorophyll content in leaf 8 of UBQ Null and UBQ OE lines. The rice codon optimized sequence for *BZR1* (rco*OsBZR1*) was cloned upstream of the maize *UBIQUITIN* promoter (p*ZmUBI*) and transformed into Kitaake rice to generate constitutive overexpression lines (referred to as UBQ OE). Total chlorophyll (µg/g fresh weight (FW)) from fully expanded leaf 8 was determined. Data are from 4 biological repeats for each treatment. The box plots show the median and the interquartile range (IQR) between the first and third quartiles, whiskers indicate the smallest and largest values within 1.5 × IQR from the quartiles, while points beyond this range are considered outliers. No statistically significant change ($p \leq 0.05$) in chlorophyll content between overexpressor and corresponding null line is represented by "ns" as determined by independent t-test.

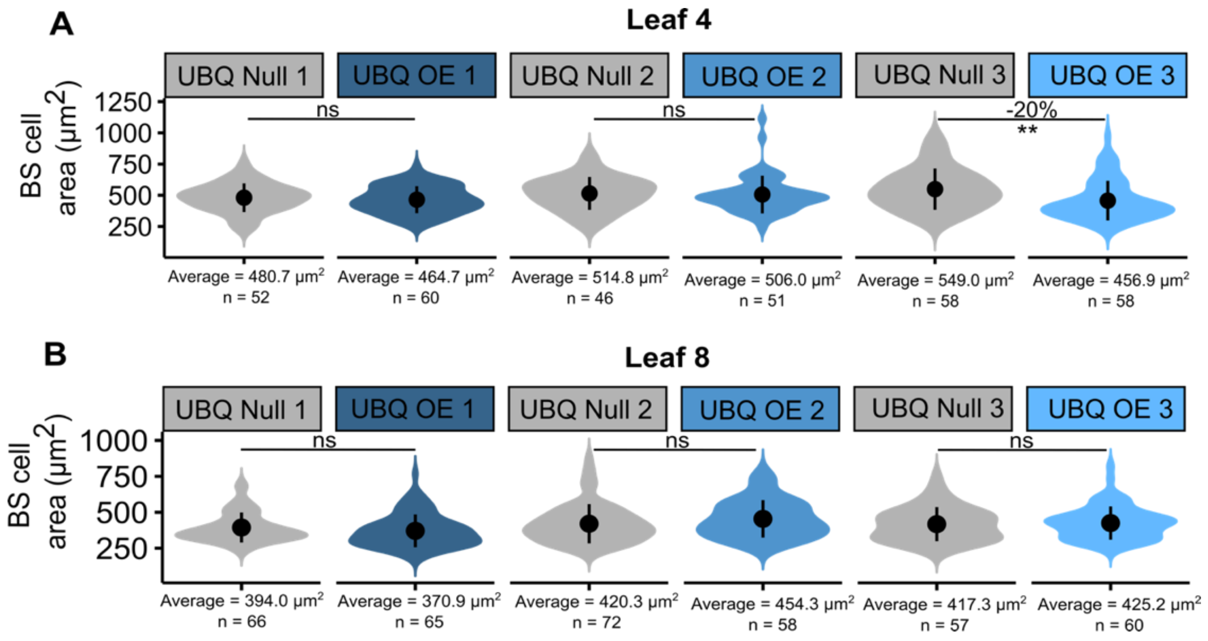

Supplemental Figure S9: Bundle sheath cell size in UBQ Null and UBQ OE lines. The rice codon optimized sequence for *BZR1* (rco*OsBZR1*) was cloned upstream of the maize *UBIQUITIN* promoter (p*ZmUBI*) and transformed into Kitaake rice to generate constitutive overexpression lines (referred to as UBQ OE). **A** and **B:** The area of individual bundle sheath (BS) cells in leaf 4 (**A**) and leaf 8 (**B**) of UBQ Null and UBQ OE plants was calculated from confocal microscope images. The center dot within each violin represents the mean chloroplast area, while the vertical bars indicate ±1 standard deviation (SD) around the mean. Percentage values above the violins indicate the change in BS cell area of UBQ OE chloroplasts compared to the corresponding null line. No statistically significant change in BS cell area is represented by “ns”. The average below each violin is the mean BS cell area calculated for that line and n represents the number of BS cells quantified for this average. Four biological replicates were used for each line. Stars above violins indicate a statistically significant difference between UBQ OE and corresponding null line as determined by independent t-test, where $p \leq 0.05$ is flagged with one star (*), $p \leq 0.01$ is flagged with 2 stars (**) and $p \leq 0.001$ is flagged with three stars (***).

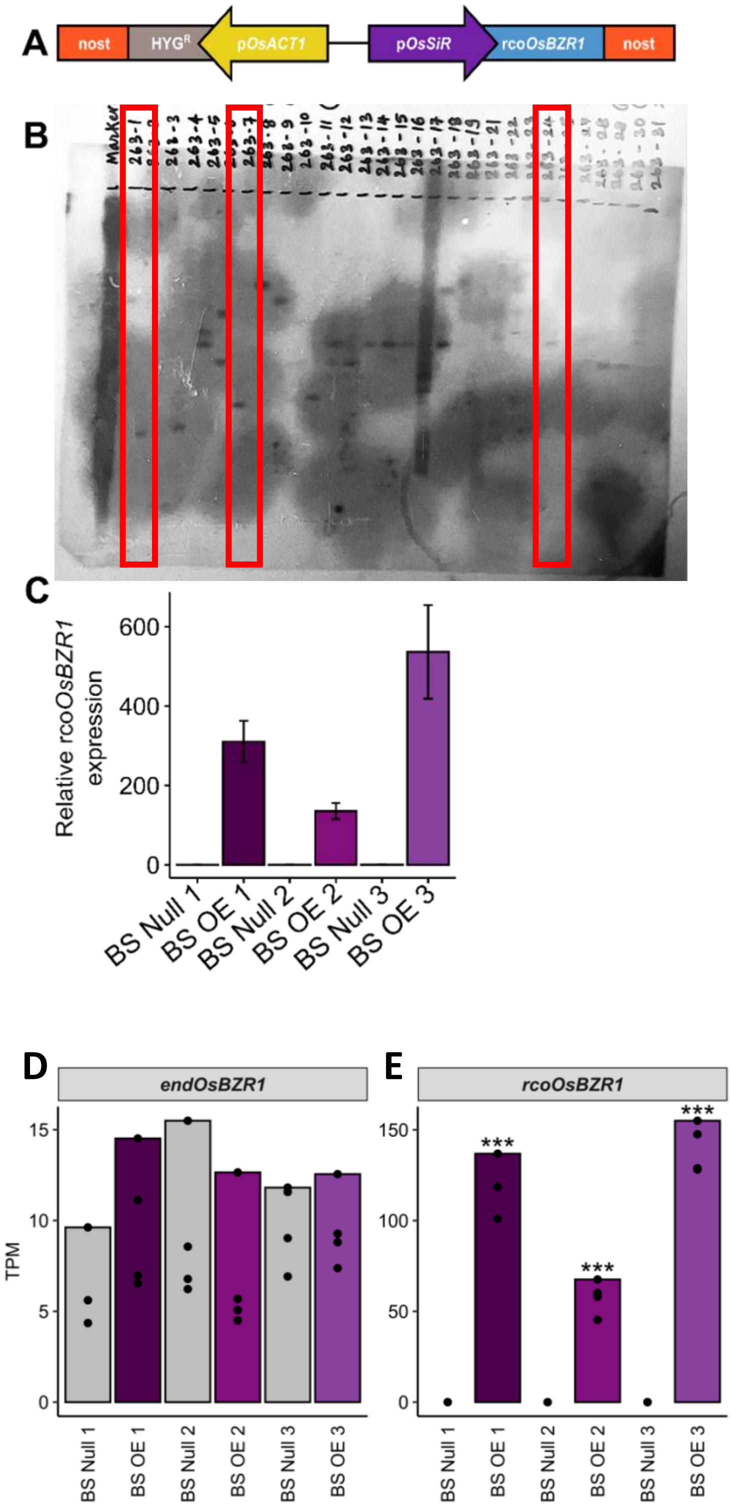

Supplemental Figure S10: Development of rice lines overexpressing rco*OsBZR1* specifically in bundle sheath cells (BS OE). A: Schematic of the construct used for transformation. The hygromycin resistance gene (HYG^R) driven by the ACTIN promoter (p*OsACT1*) was used to select transformants. The rice *SULFITE REDUCTASE* promoter

(p*OsSiR*) was used to drive bundle sheath cell-specific expression of rice codon optimized BZR1 (rco*OsBZR1*). **B:** Southern blot performed on DNA from T_0 transformants to identify lines containing single copies of the T-DNA insert. Red boxes outline the three independent, single copy lines chosen for phenotyping. 263-1, 263-26 and 263-7 refer to BS OE 1, BS OE 3 and BS OE 2 respectively. **C:** Homozygous lines in the T_2 generation were identified and the level of rco*OsBZR1* expression in leaf 4 determined by RT-qPCR using rco*OsBZR1* specific primers. Expression is shown relative to *OsEF1α* and *OsUBI5* reference genes and the mean from four biological replicates presented. Error bars represent standard errors. **D** and **E:** Endogenous (end*OsBZR1*) and rice codon optimized (rco*OsBZR1*) *OsBZR1* transcript abundance (transcripts per million – TPM) in homozygous T_2 plants was determined by bulk RNA sequencing**.** Data represents the mean derived from four biological replicates. Stars above bars indicate a statistically significant difference between BS OE and corresponding null line as determined by independent t-test, where $p \leq 0.05$ is flagged with one star (*), $p \leq 0.01$ is flagged with 2 stars (**) and $p \leq 0.001$ is flagged with three stars (***).

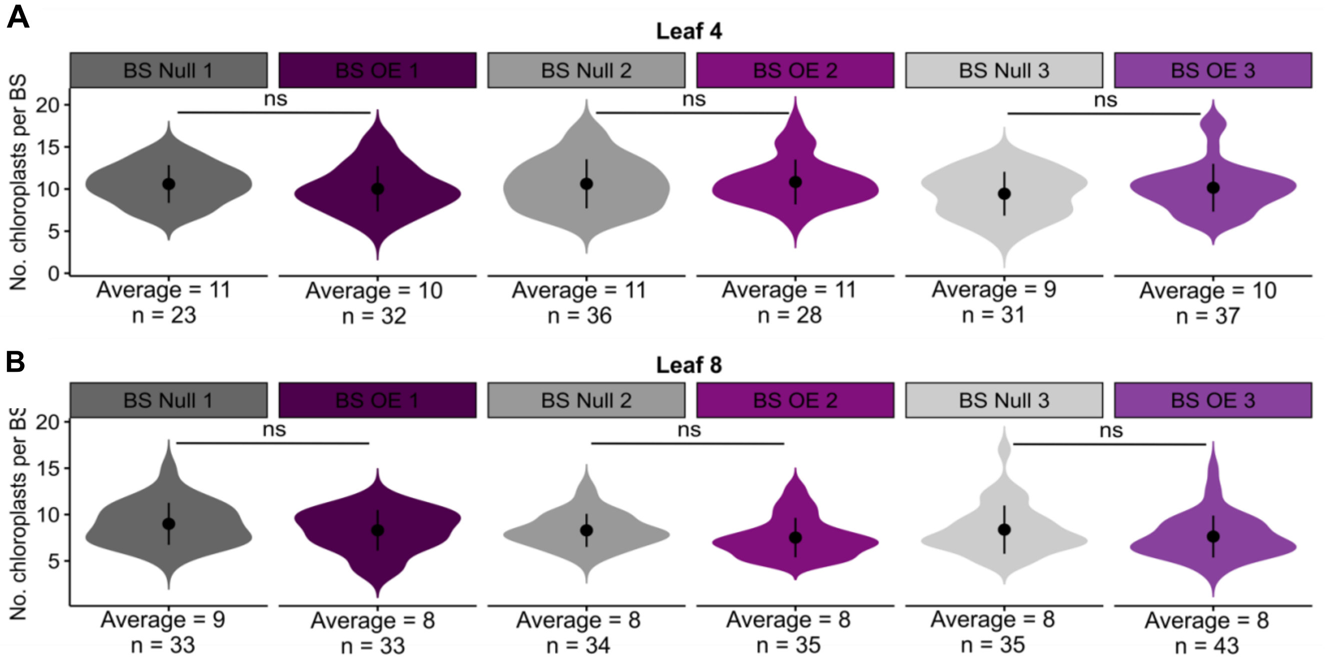

Supplemental Figure S11: Bundle sheath cell-specific overexpression of *OsBZR1* does not impact chloroplast number per bundle sheath cell. The rice codon optimized sequence for *BZR1* (rco*OsBZR1*) was cloned upstream of the rice bundle sheath cell-specific *SULFITE REDUCTASE* promoter (p*OsSIR*) and transformed into Kitaake rice to generate cell-specific overexpression lines (referred to as BS OE). **A** and **B:** The number of chloroplasts per bundle sheath cell (BSC) in leaf 4 (**A**) and leaf 8 (**B**) was quantified from the confocal images. The center dot within each violin represents the mean number of chloroplasts per bundle sheath cell, while the vertical bars indicate ±1 standard deviation (SD) around the mean. The average below each violin is the mean number of chloroplasts per bundle sheath cell calculated for that line and n represents the number of BS cells assessed. Four biological replicates were used for each line. No statistically significant change ($p \leq 0.05$) in the number of chloroplasts per bundle sheath cell of BS OE chloroplasts compared with corresponding null line is represented by “ns” as determined by independent t-test.

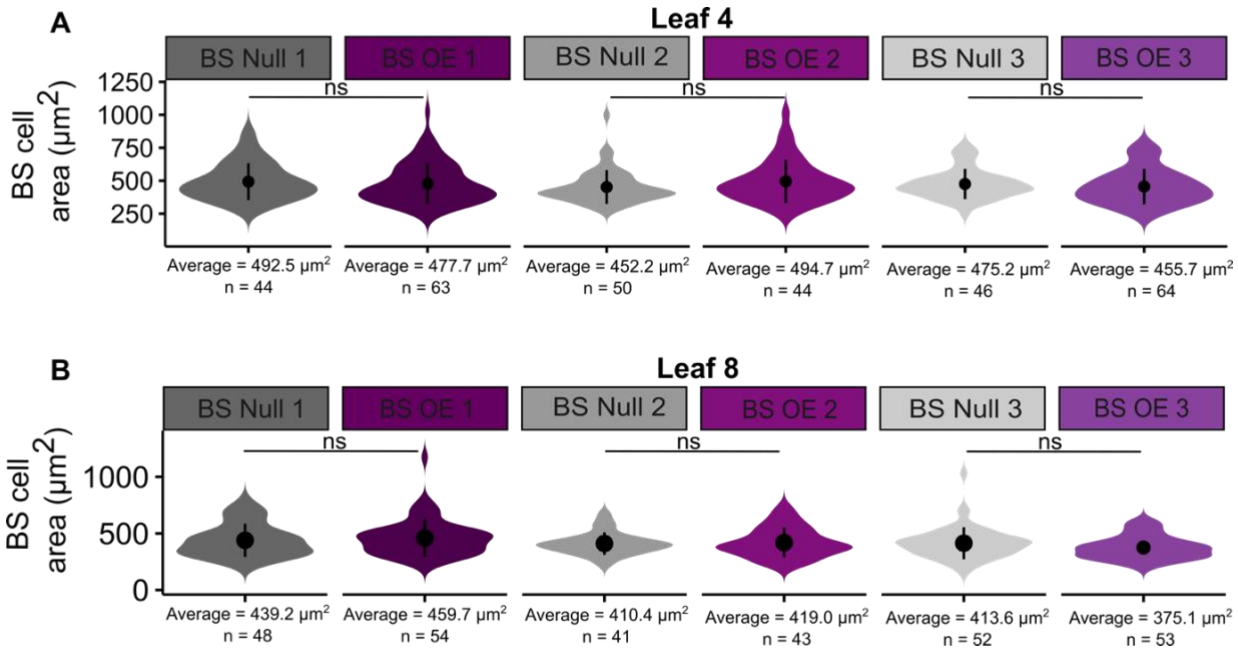

Supplemental figure S12: Bundle sheath cell-specific overexpression of *OsBZR1* does not impact bundle sheath cell size. The rice codon optimized sequence for *BZR1* (rco*OsBZR1*) was cloned upstream of the rice bundle sheath cell-specific *SULFIITE REDUCTASE* promoter (p*OsSIR*) and transformed into Kitaake rice to generate cell-specific overexpression lines (referred to as BS OE). **A** and **B:** The area of individual bundle sheath (BS) cells in leaf 4 (**A**) and leaf 8 (**B**) of BS Null and BS OE plants was determined from confocal microscopy. The center dot within each violin represents the mean chloroplast area, while the vertical bars indicate ±1 standard deviation (SD) around the mean. The average below each violin is the mean BS cell area calculated for that line and n represents the number of BS cells assessed. Four biological replicates were used for each line. No statistically significant change ($p \leq 0.05$) in BS cell area of BS OE chloroplasts compared with corresponding null line is represented by “ns” as determined by independent t-test.

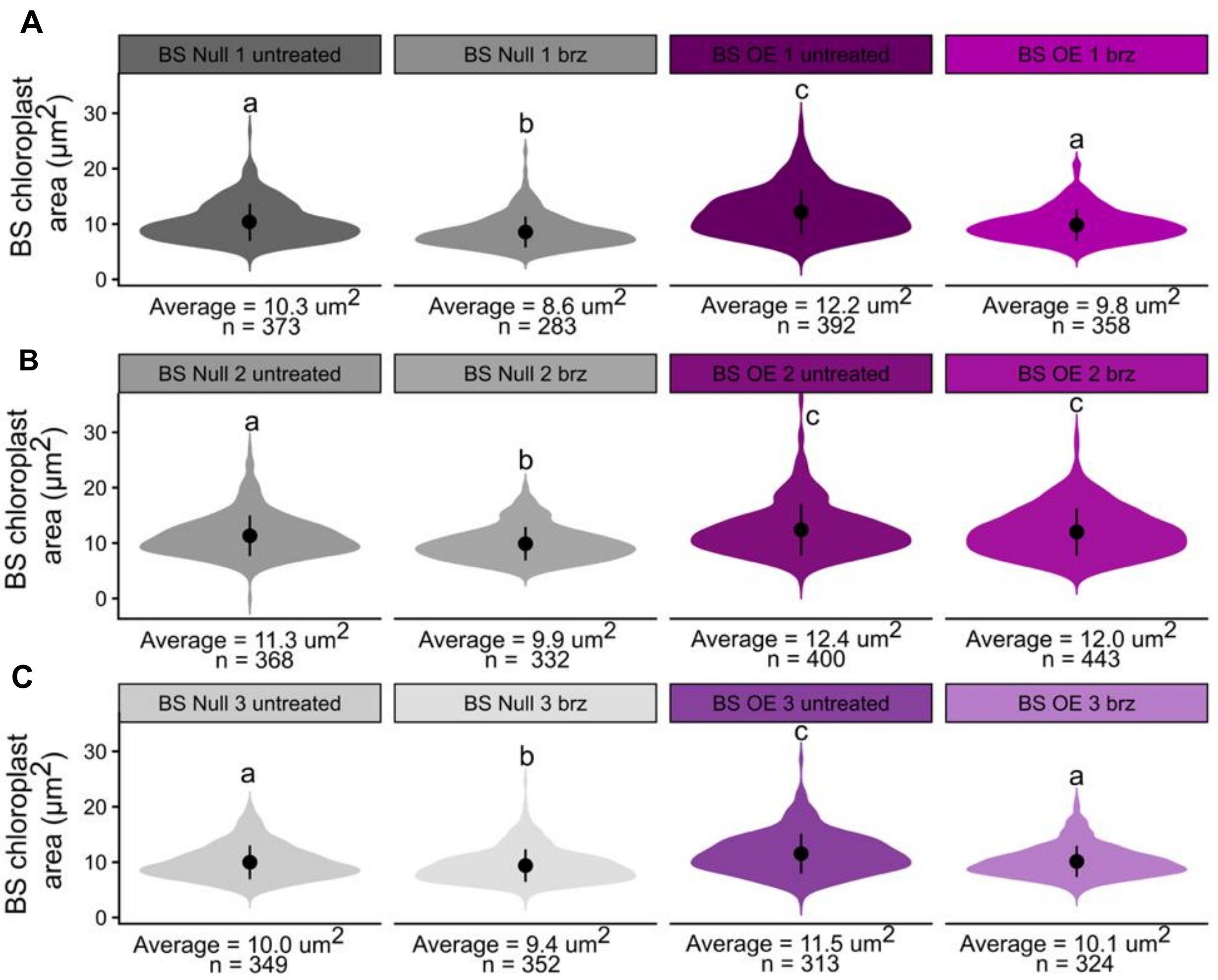

Supplemental Figure S13: Brassinazole suppresses the positive effect of bundle sheath cell-specific overexpression of *OsBZR1*. Seeds for cell-specific overexpression lines (BS OE) and corresponding null lines (BS null) were germinated in water and transferred to ½ MS-agar media with or without 10 µM brassinazole (Brz). **A**, **B** and **C:** The area of individual bundle sheath (BS) cell chloroplasts in leaf 4 of BS Null and BS OE plants was calculated from confocal microscope images. The center dot within each violin represents the mean chloroplast area, while the vertical bars indicate ±1 standard deviation (SD) around the mean. The average below each violin is the mean BS cell chloroplast area calculated for that line and n represents the number of BS chloroplasts assessed. Data are derived from confocal microscopy and from at least 300 chloroplasts for each line in each treatment. Letters above violins represent statistically significant differences ($p \leq 0.05$) in mean BS cell chloroplast area as determined by Fisher LSD post-hoc analysis following a one-way ANOVA.

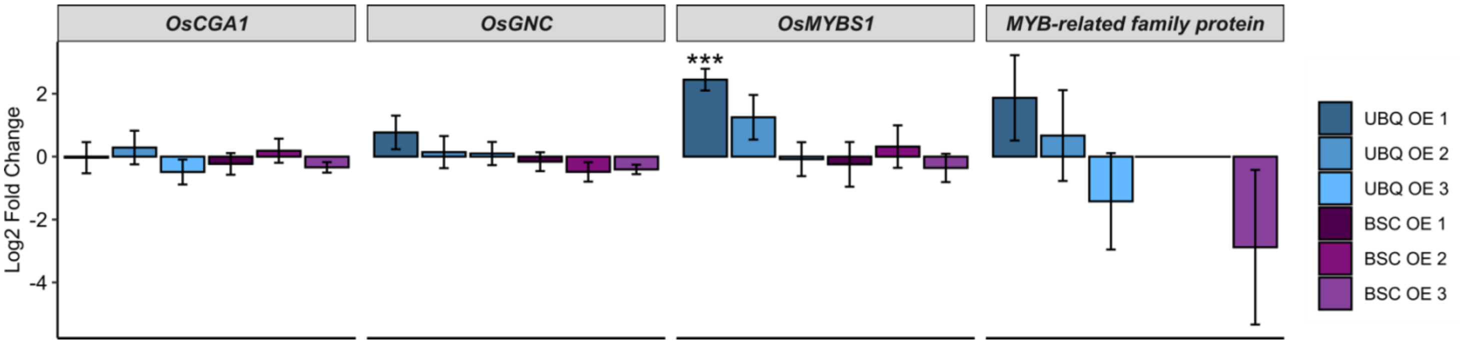

Supplemental figure S14: Expression of other master regulators of chloroplast development in the constitutive and bundle sheath cell-specific *OsBZR1* overexpressors. Log2 fold change (FC) in expression of genes involved in chloroplast development *OsCGA1*, *OsGNC*, *OsMYCS1* and MYB-related family protein. Log2 FC was determined by DESeq2 analyses using four biological replicates for each line. Statistical significance was determined using the Wald test for pairwise comparisons and *p*-values adjusted for multiple testing using the Benjamini-Hochberg (FDR) correction. Stars above bars indicate a statistically significant difference in Log2 FC expression between OE and corresponding null line as determined by independent t-test where *p*-adj. ≤ 0.05 is flagged with one star (*), *p*-adj. ≤ 0.01 is flagged with 2 stars (**) and *p*-adj. ≤ 0.001 is flagged with three stars (***). Error bars represent standard error. Refer to Supp. dataset 4 for full gene names and gene IDs.
